# Associated factors of white matter hyperintensity volume: a machine-learning approach

**DOI:** 10.1038/s41598-021-81883-4

**Published:** 2021-01-27

**Authors:** Sergio Grosu, Susanne Rospleszcz, Felix Hartmann, Mohamad Habes, Fabian Bamberg, Christopher L. Schlett, Franziska Galie, Roberto Lorbeer, Sigrid Auweter, Sonja Selder, Robin Buelow, Margit Heier, Wolfgang Rathmann, Katharina Mueller-Peltzer, Karl-Heinz Ladwig, Hans J. Grabe, Annette Peters, Birgit B. Ertl-Wagner, Sophia Stoecklein

**Affiliations:** 1 Department of Radiology, University Hospital, LMU Munich, Marchioninistraße 15, 81377 Munich, Germany; 2 Institute of Epidemiology, Helmholtz Zentrum München, German Research Center for Environmental Health, Munich-Neuherberg, Germany; 3 Biggs Institute Neuroimaging Core (BINC), Glenn Biggs Institute for Neurodegenerative Disorders, University of Texas Health Science Center At San Antonio, San Antonio, USA; 4 Department of Radiology and Penn Memory Center, Center for Biomedical Image Computing and Analytics, University of Pennsylvania, Philadelphia, USA; 5 Institute of Community Medicine and Department of Psychiatry, University of Greifswald, Greifswald, Germany; 6 Department of Diagnostic and Interventional Radiology, Medical Center - University of Freiburg, Freiburg, Germany; 7 Institute of Diagnostic Radiology and Neuroradiology, University of Greifswald, Greifswald, Germany; 8 Institute for Biometrics and Epidemiology, German Diabetes Center, Duesseldorf, Germany; 9 German Center for Diabetes Research (DZD), Munich-Neuherberg, Germany; 10 Department of Psychosomatic Medicine and Psychotherapy, Klinikum Rechts Der Isar, Technical University Munich, Munich, Germany; 11 German Center for Neurodegenerative Diseases (DZNE), Rostock, Greifswald Germany; 12 German Centre for Cardiovascular Research (DZHK E.V.), Munich, Germany; 13 Chair of Epidemiology, Ludwig-Maximilians-University München, Munich, Germany; 14 Department of Radiology, The Hospital for Sick Children, University of Toronto, Toronto, Canada; 15 KORA Study Centre, University Hospital of Augsburg, Augsburg, Germany

**Keywords:** Neurodegeneration, White matter disease, Computer science

## Abstract

To identify the most important parameters associated with cerebral white matter hyperintensities (WMH), in consideration of potential collinearity, we used a data-driven machine-learning approach. We analysed two independent cohorts (KORA and SHIP). WMH volumes were derived from cMRI-images (FLAIR). 90 (KORA) and 34 (SHIP) potential determinants of WMH including measures of diabetes, blood-pressure, medication-intake, sociodemographics, life-style factors, somatic/depressive-symptoms and sleep were collected. Elastic net regression was used to identify relevant predictor covariates associated with WMH volume. The ten most frequently selected variables in KORA were subsequently examined for robustness in SHIP. The final KORA sample consisted of 370 participants (58% male; age 55.7 ± 9.1 years), the SHIP sample comprised 854 participants (38% male; age 53.9 ± 9.3 years). The most often selected and highly replicable parameters associated with WMH volume were in descending order age, hypertension, components of the social environment (i.e. widowed, living alone) and prediabetes. A systematic machine-learning based analysis of two independent, population-based cohorts showed, that besides age and hypertension, prediabetes and components of the social environment might play important roles in the development of WMH. Our results enable personal risk assessment for the development of WMH and inform prevention strategies tailored to the individual patient.

## Introduction

White matter hyperintensities (WMH) of the brain were considered as incidental findings without clinical significance for a long time. However, several studies over the last decade have shown that WMH are associated with various severe morbidities such as stroke and cognitive decline^1–3^. Considering that WMH are more prevalent in old age and that our population is continuously aging, it is becoming increasingly important to draw a clear picture of the associated risk factors of WMH in order to identify appropriate preventive measures and early treatment strategies.

The pathophysiology and aetiology of WMH, however, is not yet fully understood. Histopathological findings in regions of WMH are myelin paleness, tissue rarefaction with loss of myelin and axons, as well as mild gliosis^4,5^. Hypoxic-ischemic axonal loss and demyelination, hypoperfusion due to altered cerebrovascular autoregulation as well as blood–brain barrier dysfunction have been established as leading pathophysiological causes for WMH^5,6^.

A wide range of potential risk factors for WMH have been described. Our targeted literature search, that included the 100 most relevant publications on WMH over the past 5 years, identified N = 45 different parameters potentially associated with WMH. Further details are presented in the Supplemental Material. Frequently described factors include hypertension^7–9^, age^1,10,11^, diabetes^12–14^, dyslipidaemia^15–17^, and renal impairment^18–20^. Yet, studies assessing the effect of these parameters on WMH show partially conflicting results. For instance, hypertension is one of the most commonly identified factors associated with WMH^1,7–9^. It was demonstrated that adequate antihypertensive treatment may reduce the course of WMH progression^8,9^. On the other hand, from age 73 to 76 years neither measured blood pressure nor self-reported hypertension were significant predictors of WMH volume change^17^. Wardlaw et al. showed in 2 prospective cohorts that vascular risk factors including hypertension explained only 0.1–2.0% of WMH variance, suggesting that “nonvascular” factors largely contribute to the aetiology of WMH^21^. As another example, studies assessing the effect of diabetes status on WMH show inconsistent results. While some studies demonstrated an association of WMH burden with diabetes, but not prediabetes^12,13^, a recent population-based cohort study showed a significant association of WMH burden with prediabetes and further WMH increase in type 2 diabetes^14^. These results might be partially explained by strong collinearity and partially small effect size of many potential risk factors.

Regarding the plethora of parameters influencing the development of WMH, drawing a clear picture of the most powerful parameters associated with WMH volume is challenging, especially as these factors usually do not occur isolated but as a combination of multiple risk factors determining WMH volume. In this context, classic regression models have major downsides. First, they are inherently hypothesis-driven, requiring prior knowledge about relevant confounding variables. Second, they are unable to deal with multiple correlated, possibly collinear variables. Third, they have limitations in datasets with a large number of variables in relation to sample size. Thus, more complex methods are required to thoroughly evaluate predictors of WMH. Elastic net regularization is a machine-learning method able to select and rank the most important factors out of a large number of variables, even when intercorrelation is present^22^. For instance, elastic net regularization was previously applied to identify the most important parameters associated with gray matter volume, showing better performance than a constant linear regression model^23^.

The aim of this study was to use elastic net machine-learning algorithms in order to disentangle and better understand the respective roles of different variables on WMH volume in two independent population-based cohorts.

## Methods

### Study design

Our study comprises cross-sectional data from two independent, population-based cohorts. The MRI substudy of the Cooperative Health Research in the Region of Augsburg (KORA) study served to train and internally test the elastic net regression model^24^. This yielded a “top 10 list” of important predictors of WMH volume, ranked according to their relative importance. The MRI substudy of the Study of Health in Pomerania (SHIP-TREND-0) was used as a second independent sample to externally assess the robustness of these predictors of WMH volume^25^.

KORA-MRI was sampled in Southern Germany and consists of 400 participants who underwent whole-body MRI to investigate differences in subclinical disease between diabetic, prediabetic and normoglycemic participants. Therefore, the study was enriched with participants with impaired glucose metabolism, i.e. 54 individuals (13.5%) had established Type 2 diabetes and 103 individuals (25.8%) had prediabetes^26^. SHIP-TREND-0 was sampled in North-eastern Germany and comprises 2188 MRI participants. There was no enrichment for impaired glucose metabolism. Further details of the respective study designs are presented in the Supplementary Material.

This study was approved by the ethics committee of the Bavarian Chamber of Physicians, the ethics committee of the Ludwig-Maximilians-University Munich and the ethics committee of the University of Greifswald and complies with the Declaration of Helsinki, including written informed consent of all participants.

### Collected parameters

As potential predictors of WMH volume, a panel of 90 parameters including measures of diabetes, blood pressure, adipose tissue, medication intake, sociodemographics including gender, anthropometrics, life-style factors, somatic and depressive symptoms, and sleep were collected in a standardised method as part of the KORA study design and have been described previously^24^.

Briefly, the applied definition of hypertension was systolic blood pressure ≥ 140 mmHg, diastolic blood pressure ≥ 90 mmHg and/or use of antihypertensive medication, given that the individuals were aware of being hypertensive. “Hypertension, unknown” was defined as unawareness of hypertension in a participant with hypertension, “controlled hypertension” as self-reported diagnosis of hypertension by a physician and intake of antihypertensive medication. Medications were classified as antihypertensive if the compounds were regarded as antihypertensively effective by recent guidelines. Diabetes was determined as either established type 2 diabetes validated by a physician or by fasting glucose level and OGTT. For the definition of prediabetes and diabetes the World Health Organization/International Diabetes Federation criteria were applied^27^. HbA_1c_ values were assessed. Details on the collected parameters are provided in Supplementary Table 2.

### MRI

In the KORA sample, image acquisition was performed on a single 3 T MRI system (Magnetom Skyra; Siemens AG, Healthcare Sector, Erlangen, Germany). WMH volume was assessed on T2w 3D-FLAIR sequences (SPACE, slice thickness (ST): 0.9 mm, 0.5 mm × 0.5 mm in-plane spatial resolution, repetition time (TR): 5000 ms, echo time (TE): 389 ms, inversion time (TI): 1800 ms, flip angle: 120°), in accordance with STRIVE recommendations^28^.

In the SHIP sample, imaging was performed on a single 1.5 T MRI system (Magnetom Avanto; Siemens AG, Healthcare Sector, Erlangen, Germany). WMH volume was assessed on T1w sequences (ST: 1.0 mm, 1 × 1 mm in-plane spatial resolution, TR: 1900 ms, TE: 3.4 ms, flip angle: 15°) and T2w 3D-FLAIR sequences (ST: 3.0 mm, 0.9 × 0.9 mm in-plane spatial resolution, TR: 5000 ms, TE: 325 ms, flip angle: 15°), in accordance with STRIVE recommendations^28^.

### WMH volume

**Figure 1:**
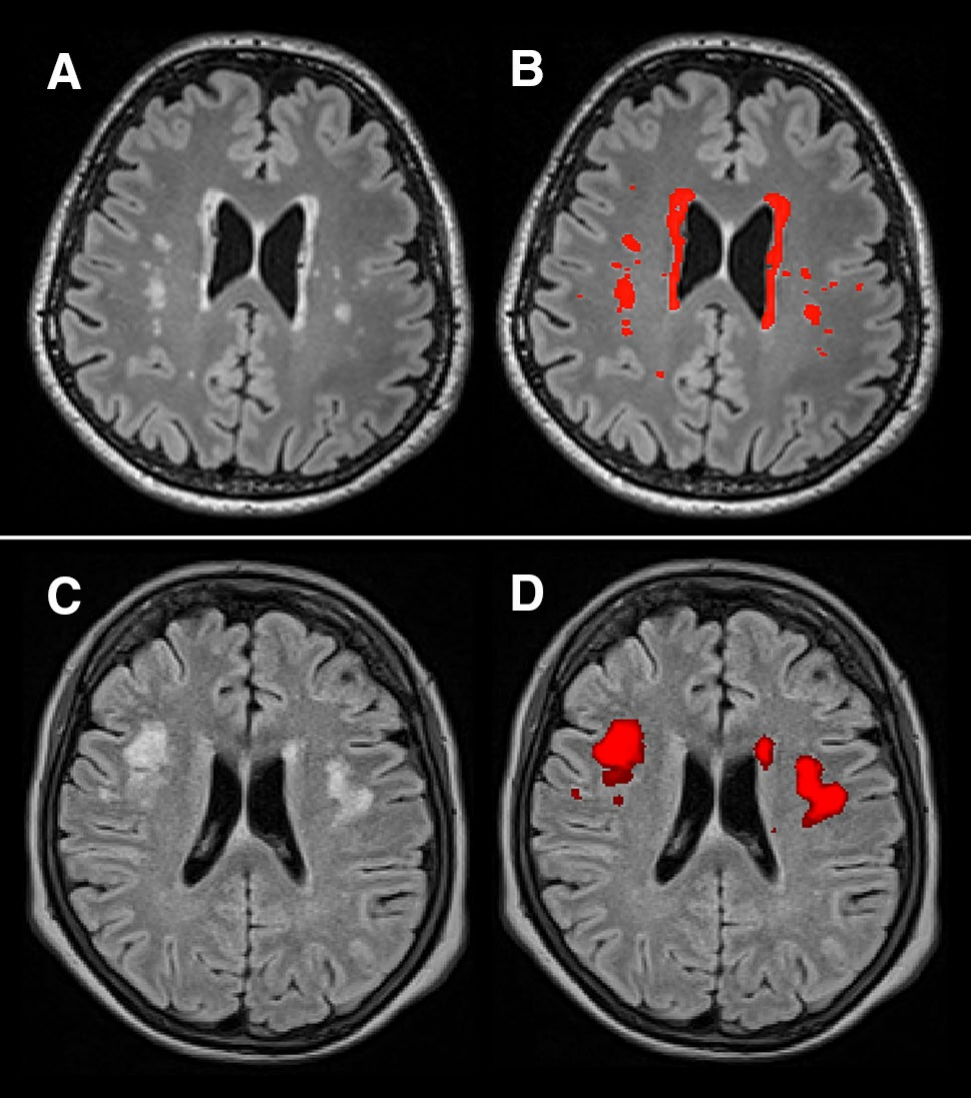
Example of manual WMH segmentation in the KORA sample (**A**,**B**) and automated WMH segmentation in the SHIP sample (**C**,**D**). (**A**) Axial view of the T2-weighted fluid attenuated inversion recovery data of a KORA participant with a WMH volume of 9600 mm^3^. (**B**) Manual segmentation of WMH in KORA. (**C**) Axial view of the T2-weighted fluid attenuated inversion recovery data of a SHIP participant with a WMH volume of 8600 mm^3^. (**D**) Automated multimodal segmentation of WMH in SHIP.

In the KORA sample, ITK-SNAP Version 3.6.0 was used for segmentation^29^. Cerebral WMH were manually segmented by a radiology resident (2 years of experience in neuroimaging), and edited and modified where necessary by a board-certified radiologist (7 years of experience in neuroimaging) on sagittal acquired FLAIR images reconstructed in axial plane with a ST of 0.5 mm (see Fig. 1). For homogeneous image intensity the tool “auto-adjust contrast” in ITK-SNAP Version 3.6.0 was used^29^. WMH were defined as signal abnormalities of variable size in the white matter of the brain that show a hyperintense signal on FLAIR images^28^. WMH in the brainstem and the cerebellum were not included.

In the SHIP sample an automated multimodal segmentation algorithm for WMH quantification was used. The algorithm produced a probabilistic map that was further thresholded to generate a binary image^3^. Furthermore, to calculate WMH volume within specific regions of interest a multiatlas segmentation method was applied^30^. This included nonlinear registration of multiple atlases with ground-truth labels for every individual scan. Finally, WMH volume was determined for every region of the brain by masking WMH from all other regions^31^. For the present analysis, measurements from the frontal, parietal, temporal and occipital lobes were summarised.

Image analyses were performed blinded to all clinical data as well as other measurements.

### Descriptive statistics

Continuous predictor covariates are described as arithmetic means with standard deviation (SD) or medians with 1st and 3rd quartile. Categorical predictor variables are presented as counts and percentages. P-values < 0.05 are considered to denote statistical significance.

### Analysis model

In both the KORA and SHIP sample, the outcome of interest was WMH volume on a continuous scale. We identified relevant predictor covariates associated with WMH volume in the KORA cohort by penalised zero-inflated negative binomial (ZINB) regression models based on elastic net regularization^32^. The ZINB model accounts simultaneously for the skewed distribution of WMH volumes with overdispersion (“count part”) and the large point mass at zero stemming from those participants without WMH (“structural zeros”). Elastic net combines the properties of both Ridge and least absolute shrinkage and selection operator (LASSO) regression and is therefore appropriate for variable selection on potentially correlated covariates^22^. The amount of blending between Ridge and LASSO regression is regulated by the hyperparameter α (Ridge: α = 0, LASSO: α = 1). All analyses were computed on a grid of α values from 0.01 to 1 with stepwise increments of 0.1.

The model was derived and evaluated on 1000 data splits. A data split was defined as a random division of the full data set into 90% training data and 10% testing data. By evaluating the model on 1000 data splits with mutually exclusive training and testing data, we ensured a very comprehensive internal model validation. Continuous covariates were standardised to mean = 0 and SD = 1. A ZINB model was computed on the training data, with WMH volume data being modelled by a negative binomial model using logarithmic link. The shrinkage parameter λ was determined by internal tenfold cross validation on the training data with upper thresholds being fractions of 0.5 and 0.1 of λ_max_ (the smallest value of λ for which all coefficients are shrunk to zero). Selection frequency across the 1000 splits served as a measure of variable importance. To disentangle and assess the roles of different variables in their association with WMH volume, both the model’s explanatory performance and its predictive performance have to be evaluated. Root Mean Squared Error (RMSE) served as a measure of predictive performance on the testing data and Akaikes Information Criterion (AIC) served as measures of model fit, i.e. explanatory performance, on the training data. Coefficient estimates are reported as raw beta values which have to be exponentiated to obtain incidence rate ratios. For comparison, we calculated the Null ZINB model that includes no covariates and predicts the mean WMH volume for each participant. A likelihood-ratio test was used to formally assess the model fit of the final model compared to the Null model.

Predictors of WMH volume identified in the KORA sample were then ranked according to selection frequency across 1000 data splits. A cut-off value based on selection frequency was not applicable due to the varying numbers of parameters in the KORA sample (N = 90) and SHIP sample (N = 34). Consequently, the ten most frequently selected variables were subsequently examined in the SHIP sample. A negative binomial regression model was evaluated on the whole sample and compared to the Null model predicting constant WMH volume in terms of RMSE and AIC. Furthermore, analogous to the procedure on the KORA cohort, variables were evaluated on 1000 data splits with 90% training and 10% testing data and ranked according to selection frequency.

As a sensitivity analysis, the complete machine-learning pipeline was re-run on a subsample of N = 333 participants of the KORA study with available data on intracranial volume (ICV), including ICV as an additional predictor to all the other variables.

R version 3.4.4 was used for all statistical analyses, including descriptive statistics. Package zipath 0.3–5 was used for calculation of ZINB models.

## Results

### Study population

**Figure 2:**
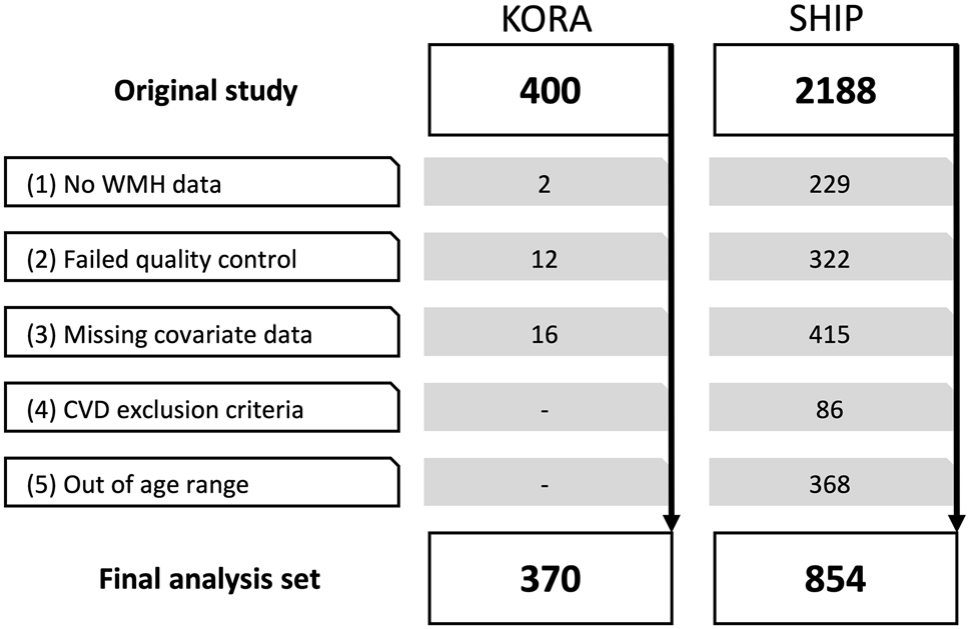
Participant flow diagram of the KORA sample and SHIP sample. (1): Missing WMH volume data or visible lesions with other aetiology. (2): Insufficient image quality (3): KORA: missing visceral or hepatic fat measurements. SHIP: missing OGTT. (4): CVD exclusion criteria were prior myocardial infarction, stroke or revascularization. KORA participants fulfilling these criteria were not eligible for MRI by study design^26^. For consistency between the two studies SHIP participants fulfilling these criteria were excluded. (5): KORA-MRI participants were between 39 and 73 years old by study design^26^. For consistency SHIP participants outside this age range were excluded.

**Figure 3:**
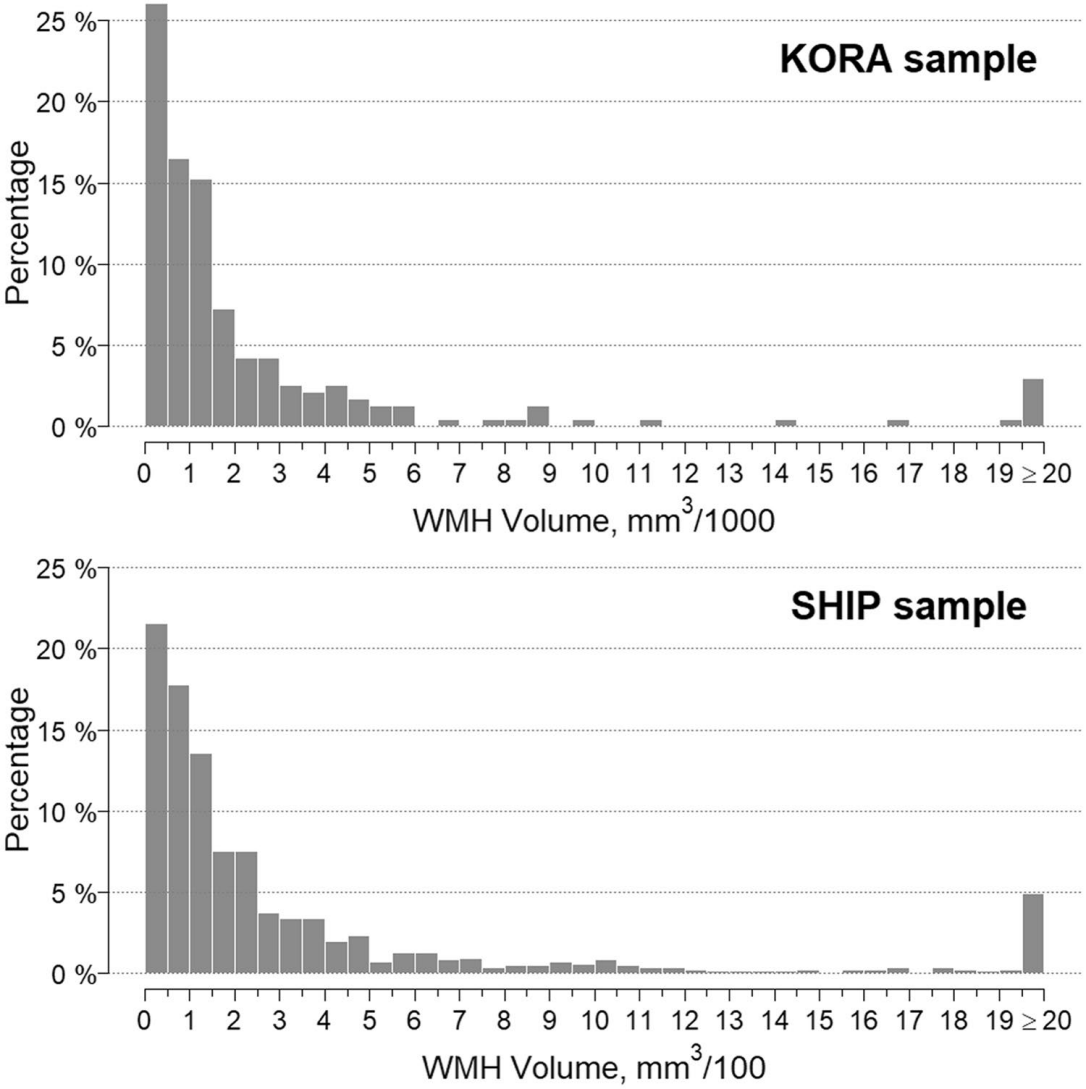
Categorised distribution of WMH volume in the KORA sample and SHIP sample. Mean WMH volume was 2798 ± 7392 mm^3^ (median: 997 mm^3^) in KORA and 532.4 ± 1750.0 mm^3^ (median 135.5 mm^3^) in SHIP. Possible explanations for this discrepancy in WMH volume are different measurement methods and different study collectives. Note that for elastic net regression, data was modelled continuously and not categorised.

**Figure 4:**
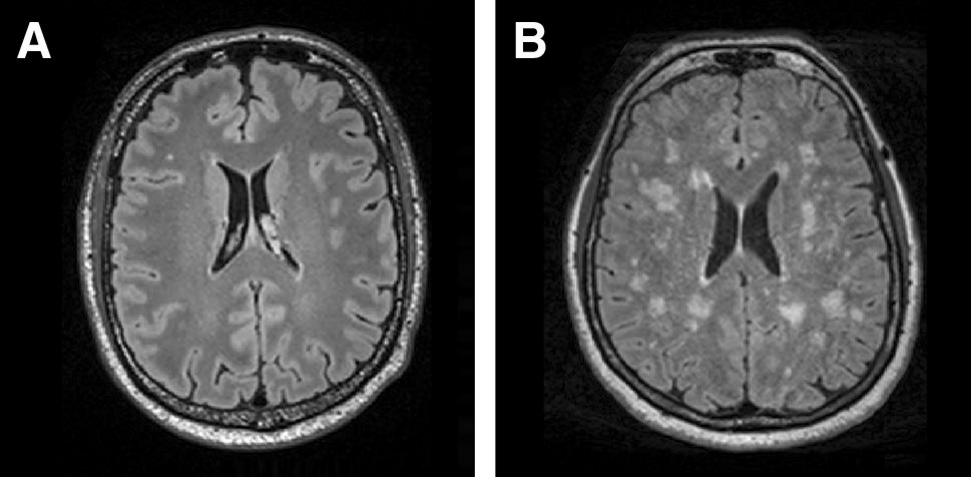
Example of different WMH volumes. Axial view of the T2-weighted fluid attenuated inversion recovery data of a subject with low WMH volume of 58 mm^3^ (**A**) and of a subject with high WMH volume of 31,300 mm^3^ (**B**).

In the KORA sample of 400 participants 12 had to be excluded due to insufficient MRI image quality, 2 due to visible lesions with other aetiology (1 participant with lesions suspicious for multiple sclerosis; 1 participant with not WMH-like FLAIR-hyperintense lesion in the left parietal lobe) and 16 participants due to missing covariate data. The final KORA sample consisted of 370 participants (58% male; age: 55.7 ± 9.1 years). In the SHIP sample of 2188 participants 229 had to be excluded due to missing WMH data, 322 due to insufficient MRI image quality, 415 due to missing covariate data. In addition, for consistency between the KORA and SHIP study 86 SHIP participants with prior myocardial infarction, stroke or revascularization and 368 SHIP participants younger than 39 years or older than 73 years were excluded. The final SHIP sample consisted of 854 participants (38% male; age 53.9 ± 9.3 years), as presented in Fig. 2. Further details are presented in Table 1. In the KORA sample, mean WMH volume was 2798 ± 7392 mm^3^ (median: 997 mm^3^) compared to 532 ± 1750 mm^3^ (median: 135 mm^3^) in the SHIP sample. The distribution of WMH volume is presented in Fig. 3, an example of different WMH volumes in Fig. 4.

**Table 1:** Description of predictor covariates for the KORA sample and SHIP sample.

KORA sample		SHIP sample		p
N = 370		N = 854		
**Sociodemographics**		**Sociodemographics**		
Age, years	55.7 ± 9.1	Age, years	53.9 ± 9.3	0.001
Men	214 (57.8%)	Men	325 (38.1%)	< 0.001
Family status		Family status		0.040
Married, living with partner	268 (72.4%)	Living with partner	712 (83.4%)	
Unmarried, living alone	34 (9.2%)	Living alone	53 (6.2%)	
Unmarried, living with partner	15 (4.1%)			
Married, not living with partner	9 (2.4%)			
Divorced	29 (7.8%)	Divorced	61 (7.1%)	
Widowed	15 (4.1%)	Widowed	28 (3.3%)	
Schooling		Schooling		
Lower secondary school	168 (45.4%)	Lower secondary school	Not assessed	
Secondary school	86 (23.2%)	Secondary school	Not assessed	
Higher secondary school	116 (31.4%)	Higher secondary school	Not assessed	
Schooling, years	12.3 ± 2.6	Schooling, years	Not assessed	
Highest professional degree		Highest professional degree		
No degree	14 (3.8%)	No degree	Not assessed	
Apprenticeship	189 (51.1%)	Apprenticeship	Not assessed	
Vocational/technician/master craftsman	92 (24.9%)	Vocational/technician/master craftsman	Not assessed	
Engineering/polytechnic degree	4 (1.1%)	Engineering/polytechnic degree	Not assessed	
University degree	71 (19.2%)	University degree	Not assessed	
Per-capita income, Euro	1374.4 ± 712.2	Per-capita income, Euro	Not assessed	
Equivalence income, Euro	1521.0 ± 718.8	Equivalence income, Euro	Not assessed	
Social stratum, Helmert scale	15.7 ± 5.0	Social stratum, Helmert scale	Not assessed	
**Anthropometric measurements**		**Anthropometric measurements**		
Weight, kg	82.7 ± 15.9	Weight, kg	79.5 ± 15.1	< 0.001
Height, cm	171.8 ± 9.6	Height, cm	168.4 ± 9.0	< 0.001
BMI, kg/m2	28.0 ± 4.7	BMI, kg/m2	27.9 ± 4.5	0.923
Waist circumference, cm	98.2 ± 13.7	Waist circumference, cm	89.8 ± 12.7	< 0.001
Hip circumference, cm	106.7 ± 8.8	Hip circumference, cm	Not assessed	
Waist-To-Hip Ratio	0.92 ± 0.09	Waist-To-Hip Ratio	Not assessed	
Right-handed	341 (92.2%)	right-handed	Not assessed	
**Diabetes related measurements**		**Diabetes related measurements**		
Glycemic Status		Glycemic Status		0.060
Normal	229 (61.9%)	Normal	579 (67.8%)	
Prediabetes	90 (24.3%)	Prediabetes	192 (22.5%)	
Diabetes	51 (13.8%)	Diabetes	83 (9.7%)	
Duration of diabetes, years (median)	7.0 [5.5, 11.5]	Duration of diabetes, years (median)	Not assessed	
Fasting serum glucose, mg/dL	104.4 ± 23.2	Fasting serum glucose, mg/dL	Not assessed	
Fasting serum insulin, mg/dL	10.9 ± 6.8	Fasting serum insulin, mg/dL	Not assessed	
HbA_1c_, %	5.6 ± 0.7	HbA_1c_, %	5.3 ± 0.7	< 0.001
**Behaviour**		**Behaviour**		
Alcohol		Alcohol		< 0.001
No consumption	88 (23.8%)	No consumption	95 (11.1%)	
< 20 g/day	146 (39.5%)	< 20 g/day	669 (78.3%)	
< 40 g/day	70 (18.9%)	< 40 g/day	64 (7.5%)	
> 40 g/day	66 (17.8%)	> 40 g/day	26 (3.0%)	
Alcohol consumption, g/day	18.8 ± 24.2	Alcohol consumption, g/day	Not assessed	
… Spririts, g/day	0.5 ± 1.6	… Spririts, g/day	Not assessed	
… Wine, g/day	6.7 ± 14.1	… Wine, g/day	Not assessed	
… Beer, g/day	11.6 ± 19.0	… Beer, g/day	Not assessed	
Smoking		Smoking		0.015
Never smoker	135 (36.5%)	Never smoker	386 (45.2%)	
Ex-smoker	160 (43.2%)	Ex-smoker	308 (36.1%)	
Smoker	75 (20.3%)	Smoker	160 (18.7%)	
Packyears (median)	15.0 [5.0, 29.0]	Packyears (median)	Not assessed	
Physically active	220 (59.5%)	Physically active	Not assessed	
Physical activity		Physical activity		0.402
No	94 (25.4%)	No	228 (26.7%)	
Sporadic	56 (15.1%)	Sporadic	145 (17.0%)	
Regularly, around 1 h/week	114 (30.8%)	Regularly, around 1 h/week	275 (32.2%)	
Regularly, 2 h/week	106 (28.7%)	Regularly, 2 h/week	206 (24.1%)	
**Somatic and depressive symptoms**		**Somatic and depressive symptoms**		
Angina pectoris	19 (5.1%)	Angina pectoris	Not assessed	
Sf-12 Somatic Scale	49.7 ± 7.4	Sf-12 Somatic Scale	Not assessed	
PHQ-9, points	3.0 ± 2.7	PHQ-9, points	Not assessed	
DEEX scale, points	7.3 ± 4.7	DEEX scale, points	Not assessed	
**Blood pressure**		**Blood pressure**		
Systolic BP, mmHg	120.7 ± 16.7	Systolic BP, mmHg	125.8 ± 16.5	< 0.001
Diastolic BP, mmHg	75.5 ± 10.0	Diastolic BP, mmHg	77.6 ± 9.7	0.001
Pulse pressure	71.2 ± 10.0	Pulse pressure	Not assessed	
Hypertension	124 (33.5%)	Hypertension	Not assessed	
Control and awareness of hypertension		Control and awareness of hypertension		0.001
No hypertension	246 (66.5%)	No hypertension	470 (55.0%)	
Controlled hypertension	69 (18.7%)	Controlled hypertension	192 (22.5%)	
Uncontrolled hypertension	21 (5.7%)	Uncontrolled hypertension	78 (9.1%)	
Hypertension, untreated	19 (5.1%)	Hypertension, untreated	41 (4.8%)	
Hypertension, unknown	15 (4.1%)	Hypertension, unknown	73 (8.5%)	
**Laboratory Measurements**		**Laboratory Measurements**		
Glomerular Filtration Rate, ml/min	87.0 ± 12.8	Glomerular Filtration Rate, ml/min	Not assessed	
Total cholesterol, mg/dL	217.9 ± 35.9	Total cholesterol, mg/dL	Not assessed	
HDL cholesterol, mg/dL	62.0 ± 17.4	HDL cholesterol, mg/dL	Not assessed	
LDL cholesterol, mg/dL	139.7 ± 32.5	LDL cholesterol, mg/dL	Not assessed	
Triglycerides, mg/dL	130.5 ± 83.2	Triglycerides, mg/dL	Not assessed	
Uric Acid, mg/dL	5.6 ± 1.5	Uric Acid, mg/dL	Not assessed	
Creatinine, mg/dL	0.88 ± 0.15	Creatinine, mg/dL	Not assessed	
**Adipose Tissue**		**Adipose Tissue**		
Hepatic Fat, %	8.6 ± 7.8	Hepatic Fat, %	Not assessed	
Visceral Fat, l	4.5 ± 2.7	Visceral Fat, l	Not assessed	
**Medication Intake**		**Medication Intake**		
Antidiabetic	29 (7.8%)	Antidiabetic	Not assessed	
Antihypertensive	90 (24.3%)	Antihypertensive	Not assessed	
Anticoagulant	6 (1.6%)	Anticoagulant	Not assessed	
Antiplatelet	14 (3.8%)	Antiplatelet	39 (4.6%)	0.642
Thyroidal	62 (16.8%)	Thyroidal	Not assessed	
NSAID	8 (2.2%)	NSAID	73 (8.5%)	< 0.001
ASS 100/300	13 (3.5%)	ASS 100/300	Not assessed	
**Sleep**		**Sleep**		
Sleep, h/day	7.1 ± 1.1	Sleep, h/day	Not assessed	
Problems falling asleep		Problems falling asleep		
Never	237 (64.1%)	Never	Not assessed	
Sometimes	96 (26.0%)	Sometimes	Not assessed	
Often	37 (10.0%)	Often	Not assessed	
Problems keeping asleep		Problems keeping asleep		
Never	166 (44.9%)	Never	Not assessed	
Sometimes	127 (34.3%)	Sometimes	Not assessed	
Often	77 (20.8%)	Often	Not assessed	
Feeling tired and exhausted because of sleep problems		Feeling tired and exhausted because of sleep problems		
Never	236 (63.8%)	Never	Not assessed	
Sometimes	111 (30.0%)	Sometimes	Not assessed	
Often	23 (6.2%)	Often	Not assessed	

Continuous variables are presented as arithmetic means with standard deviation or medians with 1st and 3rd quartile. Categorical variables are presented as counts and percentages. P-values from t-test, Kruskal–Wallis test or χ2-test, where appropriate.

### Identification of predictors of WMH volume—KORA sample

**Figure 5:**
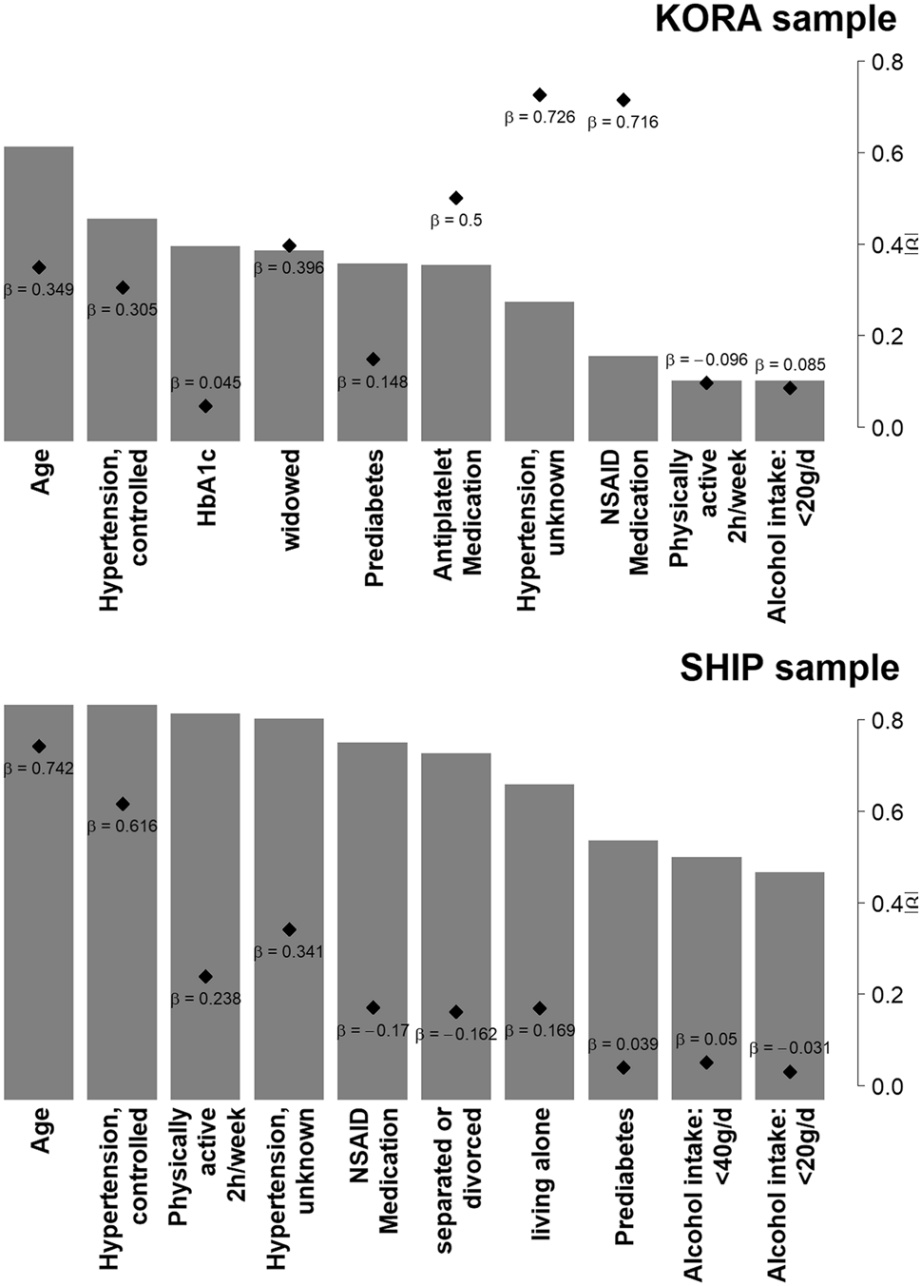
Top ten selected variables from elastic net regression in the KORA sample and SHIP sample. Results in KORA are based on α = 0.8 and a zero-inflated negative binomial model, results in SHIP based on α = 1 and a negative binomial model. On the x-axis: Predictor variable. Grey bars indicate the selection frequency (%) of the respective variable in 1000 data splits (scale according to the left y-axis). Diamonds indicate the size of the β coefficient of the respective variable, averaged over all splits where the variable was selected (scale according to the right y-axis).

In the KORA sample, the best model in terms of RMSE and AIC was obtained for α = 0.8 (RMSE = 4742 mm^3^, AIC = 4130). For comparison, the Null elastic net model (without adjusting for any cofactors) yielded RMSE = 4829 mm^3^ and AIC = 1,396,092, which serves as a proof-of-concept that the elastic net model provides additional explanatory value of WMH volume. The likelihood ratio test showed that the final model fitted the data significantly better than the Null model (p < 0.001). The ten most frequently selected variables for α = 0.8 are presented in Fig. 5. Variables included “age” (22.4% selection frequency), “controlled hypertension” (16.9%), “HbA_1c_” (14.8%), “widowed” marital status (14.5%), “prediabetes” assessed by OGTT (13.5%), “antiplatelet medication” (13.4%), “hypertension, unknown” defined as hypertension unawareness of a participant with hypertension (10.6%), “NSAID medication” (6.5%), “physical activity of 2 h/week” (4.6%; negative mean beta) and “alcohol consumption up to 20 g/day” (4.6%). Variable selection was relatively stable across all values of α, as presented in Supplementary Figure 1. In the sensitivity analysis including only KORA participants with available ICV volume, ICV was not selected in the top ten of predictors (Supplementary Table 4).

### Robustness testing of predictors of WMH volume—SHIP sample

In the SHIP sample, a negative binomial model incorporating the top ten predictors from the KORA sample yielded a RMSE of 1667 mm^3^ and an AIC of 11,600 on the whole cohort, whereas the Null model (predicting constant WMH volume) yielded an RMSE of 1749 mm^3^ and an AIC of 12,000 on the whole cohort.

When evaluating the elastic net regression model on 1000 data splits, the best model in terms of RMSE and AIC was obtained for α = 1 (RMSE = 1499 mm^3^, AIC = 10,443). For both KORA and SHIP, prediction seemed to be worse for high WMH volumes, i.e. on average the model underestimated true WMH volumes (Supplemental Figure 5). Ranking of the variables according to selection frequency is presented in Fig. 5 and Supplementary Figure 4. “Age” (selection frequency 100%), “controlled hypertension” (100%), “unknown hypertension” (97%) and “prediabetes” (66%) were replicated as important predictors. Furthermore, while “widowed” family status was not replicated in the SHIP sample, “separated or divorced” (87%) and “living alone” (80%) were selected. Other variables were either not replicated (HbA_1c_, antiplatelet medication) or showed different effect directions compared to the KORA sample (alcohol consumption, physical activity, NSAID medication).

## Discussion

In a population-based sample, we performed a data-driven analysis without a-priori hypotheses including 90 different parameters in order to disentangle and better understand the respective roles of these parameters on WMH volume. Relevant parameters were re-examined in an independent population-based sample.

Considering that WMH are associated with cognitive decline, increased stroke risk and worse outcome post stroke, decreased mobility due to gait disturbance as well as increased risk of depression, having a clear picture of the associated risk factors is important, especially regarding treatment and prevention^2,33–37^. Although a lot of information is nowadays available on the epidemiology and risk factors of WMH, some of these data are conflicting^1^. Given that plenty partially inter-correlated factors with potentially small effect size impact WMH volume, drawing a clear picture of the most powerful determinants of WMH volume is challenging. In order to overcome limitations of traditional regression models we used a machine-learning approach that allows for the selection and ranking of the most important factors out of a large number of variables, even when intercorrelation is present. Our machine-learning based model identified age and hypertension as well established determinants of WML volume. Additionally, the model identified the less established, more controversial parameters “prediabetes”, “HbA_1c_”, “alcohol consumption”, “NSAID medication” and components of the social environment such as “widowed marital status” as potential factors that contribute to WMH burden. Interestingly, ICV was not selected among the ten most important predictors. We hypothesise that in our analysis the association of ICV with WMH was captured by other variables in the model.

Diabetes-related atherosclerosis appears to be an important component in the development of WMH^38,39^. However, the relation between diabetes and particularly prediabetes and WMH is under debate^1,12,14^. Studies assessing the correlation between WMH and HbA_1c_ show conflicting results^40,41^. Interestingly, our analysis yielded prediabetes, but not diabetes, as a relevant predictor of WMH volume. This result was replicated in the SHIP sample. Furthermore, HbA_1c_ was only identified in the KORA sample, but failed to replicate in the SHIP sample. These results might be due to the co-occurrence of diabetes and hypertension and the different risk factor distribution in the two studies: Individuals with diabetes in SHIP are more likely to have hypertension (prevalence of “controlled hypertension” is 41%) compared to KORA (prevalence of “controlled hypertension” is 31%). Therefore, unfavourable effects of diabetes might be superimposed by stronger effects of unfavourable blood pressure profiles.

Our results thus suggest that the prediabetic phenotype as a dynamic state between normoglycemia and type 2 diabetes represents an independent risk factor for the development of WMH. Hence, individuals with prediabetes would need more comprehensive assessments for signs of early pathophysiological changes, preventive measures and adequate treatment not only to stop the underlying development of diabetes, but also to avoid the development of WMH-associated morbidity.

The relation between WMH and alcohol consumption is still unclear. Heavy alcohol consumption might lead to a higher WMH burden through cerebrovascular effects of associated hypertension^42^. However, after correcting for hypertension, heavy alcohol did not show a significant association with WMH in previous analyses^43,44^. Prior studies also demonstrated a protective effect of moderate alcohol consumption on the development of WMH through multiple potential pathways, including anti-atherosclerotic, anti-thrombotic and anti-inflammatory mechanisms reducing the risk of cardiocerebrovascular morbidity^43,45,46^. However, while moderate alcohol consumption was associated with decreased WMH volume in the SHIP sample, it was associated with increased WMH volume in the KORA sample; heavy alcohol consumption was associated with increased WMH volume in the SHIP sample.

In both the KORA sample and the SHIP sample, “NSAID medication” was identified as a relevant predictor of WMH volume, albeit with different effect directions. NSAID medications are generally used for pain and inflammation treatment, which comprises multiple conditions, thus rendering the groups under treatment quite heterogeneous. In KORA, all participants under NSAID medication reported regular intake of their medication, as opposed to intake as needed. No distinction between regular intake and intake as needed of NSAID medication was made in SHIP. This does not only explain the difference in prevalence (2.2% in KORA vs. 8.5% in SHIP), but might indicate that participants in KORA are affected by more severe and chronic pain. Severity of pain could be a relevant determinant of WMH volume. In this regard, our findings might support findings of previous studies that report associations of pain and increased WMH burden^47^.

The elastic net model showed a strong correlation between age and WMH burden. This result is in keeping with literature suggesting that age is the most important risk factor for WMH^1,10^. WMH are a common finding in elderly people, where WMH burden increases with age^1^. As such, WMH may to some extent be part of the normal aging process of the brain, yet precise data on the burden of WMH that can be regarded as “normal” at a certain age do not exist. As populations are aging, WMH-related morbidities, such as cognitive decline and increased stroke risk will have an increasing impact on individuals and health care systems^34,35^.

Hypertension is strongly associated with and probably the most important modifiable risk factor of WMH. Several studies clearly indicate that an increased systolic as well as diastolic blood pressure favour the development of WMH^1,3,5,7–9^. Our results are consistent with these findings. The variables “hypertension, controlled” and “hypertension, unknown (hypertension unawareness of a participant with hypertension)” were both among the 10 most frequently selected variables of the elastic net model associated with WMH. The frequent selection of the variable “hypertension, controlled” in the elastic net model could be indicative of irreversible brain damage caused by micro- and macroangiopathy in the pre-treatment episode of hypertension^38^.

A meta-analysis showed that higher physical activity was cross-sectionally associated with lower WMH volume, although effect sizes were small and many studies reported null findings^48^. A recent longitudinal study and a recent intervention trial found no effect of physical activity on WMH volumes^49,50^. In our study, high physical activity was associated with decreased WMH volume in the KORA sample, but not in the SHIP sample, indicating that the role of physical activity is unstable.

“Widowed family status”, “separated or divorced” and “living alone” are components of the social environment that were revealed to be relevant predictors of WMH volume in our study. It can be hypothesised that these predictors might comprise a cluster of mental-health related factors, such as loneliness, anxiety or post-traumatic stress disorder, which were not assessed in this study^38^. The importance of social networks and stressful life events on mental well-being and health in general^51,52^, as well as the association of social economic factors with WMH have been established^53^. It was also shown that widowhood accelerates cognitive decline in cognitively normal older adults^54^. Our results support this finding, having in mind that WMH are associated with cognitive decline^3^. However, further studies are needed to clarify the association of components of the social environment with WMH volume.

The results of this study need to be interpreted in light of its limitations. The regularised regression employed here is not a causal model in the formalised sense and thus cannot identify whether the reported variables are etiologically linked to WMH volume. For observational data, different statistical tools can be used to evaluate causality, e.g. graphical models such as directed acyclic graphs, methods based on counterfactuals from the potential outcomes framework, methods based on instrumental variables such as Mendelian Randomization which emulate the design of randomised controlled trials, or structural causal models^55^. These methods, however, require prior knowledge and assumptions about the potential etiologic layout in the analysed variables. By our statistical model, we used a hypothesis-free approach without assumptions about the underlying etiologic factors. It has been emphasised, especially within the epidemiologic field, that evidence from different study designs and models should be taken into account to investigate causality^56^. We therefore believe that the results of our prediction-based analysis can provide useful starting points to inform further, more formalised, causal reasoning.

For (zero-inflated) negative binomial models as employed here, easily interpretable metrics of the proportion of outcome variance explained are not straightforward. Therefore, the relative contribution of the respective predictor variables has to be assessed by the inclusion frequencies only.

In the same vein, the regularization by elastic net and the underlying ZINB regression represent an intricate multi-layered model with complex interpretation. However, elastic net regularization is an appropriate and established method for variable selection, and the ZINB model captures the data distribution best. By presenting a ranking according to inclusion frequencies of the identified variables, we can still provide an adequate interpretation of the findings.

Furthermore, in the KORA sample mean WMH volume was significantly higher than in the SHIP sample. Possible explanations for this discrepancy in WMH volume are different measurement methods and different study collectives. However, identical methodologies across large population-based studies are not to be expected, and the fact that some parameters were consistently associated with WMH volume in both, KORA and SHIP, does show a certain robustness of the association. Further well-characterised MRI studies are needed to corroborate these findings.

## Conclusion

In conclusion, a systematic machine-learning based analysis of 90 parameters showed in two independent samples, that besides age and hypertension prediabetes and components of the social environment (i.e. widowed, living alone) might play important roles in the development of WMH. Our results therefore enable personal risk assessment for high WMH burden and prevention strategies tailored to the individual patient.

## Supplementary Information

Supplementary Information

## Abbreviations

AIC: Akaikes information criterion
DEEX: Depressed mood and exhaustion
FLAIR: Fluid-attenuated inversion recovery
KORA: Cooperative health research in the region of Augsburg
MRI: Magnetic resonance imaging
NSAID: Nonsteroidal anti-inflammatory drug
OGTT: Glucose tolerance test result
RMSE: Root mean squared error
SHIP: Study of health in Pomerania
SD: Standard deviation
WMH: White matter hyperintensities
ZINB: Zero-inflated negative binomial
