# Supplementary material for: Associated factors of white matter hyperintensity volume: a machine-learning approach
Source: Sci Rep. 2021 Jan 27;11:2325. doi: 10.1038/s41598-021-81883-4 (PMC7840689; doi:10.1038/s41598-021-81883-4)
Supplement: Supplementary file 1 — Supplementary Information [file 41598_2021_81883_MOESM1_ESM.docx]

**Associated factors of white matter hyperintensity volume - a machine learning approach**

**Supplemental Material**

Sergio Grosu^1^, Susanne Rospleszcz^2,13^, Felix Hartmann^1^, Mohamad Habes^3,4,5^, Fabian Bamberg^6^, Christopher L. Schlett^6^, Franziska Galie^1^, Roberto Lorbeer^1^, Sigrid Auweter^1^, Sonja Selder^1^, Robin Buelow^7^, Margit Heier^2^, Wolfgang Rathmann^8,9^, Katharina Mueller-Peltzer^6^, Karl-Heinz Ladwig^2,10^, Hans J. Grabe^5,7,11^, Annette Peters^2,12,13^, Birgit B. Ertl-Wagner^1,14^, Sophia Stoecklein^1^

^1^Department of Radiology, University Hospital, LMU Munich, Munich, Germany

^2^Institute of Epidemiology, Helmholtz Zentrum München, German Research Center for Environmental Health, Munich-Neuherberg, Germany

^3^Biggs Institute Neuroimaging Core (BINC), Glenn Biggs Institute for Neurodegenerative Disorders, University of Texas Health Science Center at San Antonio, San Antonio, USA

^4^Center for Biomedical Image Computing and Analytics, and Department of Radiology and Penn Memory Center, University of Pennsylvania, Philadelphia, USA

^5^Institute of Community Medicine and Department of Psychiatry, University of Greifswald, Greifswald, Germany

^6^Department of Diagnostic and Interventional Radiology, Medical Center - University of Freiburg, Freiburg, **Germany**

^7^Institute of Diagnostic Radiology and Neuroradiology, University of Greifswald, Greifswald, Germany

^8^Institute for Biometrics and Epidemiology, German Diabetes Center, Duesseldorf, Germany

^9^German Center for Diabetes Research (DZD), Munich-Neuherberg, Germany

^10^Department of Psychosomatic Medicine and Psychotherapy, Klinikum rechts der Isar, Technical University Munich, Munich, Germany

^11^German Center for Neurodegenerative Diseases (DZNE), Rostock/Greifswald, Germany

^12^German Centre for Cardiovascular Research (DZHK e.V.), Munich, Germany

^13^Chair of Epidemiology, Ludwig-Maximilians-University München, Munich, Germany

^14^Department of Radiology, The Hospital for Sick Children, University of Toronto, Toronto, Canada

**Targeted literature search on associations with WMH:**

Literature search was performed on the Pubmed search engine from January 1^st^, 2015 to August 7^th^, 2020, run on August 8^th^, 2020, restricted to human studies published in English using the search terms listed below in order to evaluate publications on factors associated with WMH. Literature search conducted in this manner yielded 448 hits. The machine learning based Pubmed search algorithm "Best Match" was used to select the 100/448 most relevant publications on WMH for further analysis ^1^.

WMH search terms (filters applied: humans):

((White matter hyperintens*) OR (white matter lesion*) OR (white matter disease*) OR (white matter change*) OR (leukoaraiosis)) AND ((leukoaraiosis) OR (cerebral small vessel disease) OR (vascular dementia)) AND (MRI) AND (risk factor*)

Our targeted literature search identified N=45 different factors potentially associated with WMH, which are listed in Supplementary Table 1 in descending order:

**Supplementary Table I:**

| hypertension (N=11) ^2-12^ |
| --- |
| age (N=10) ^4,5,10,13-19^ |
| cognitive impairment (N=6) ^6,20-24^ |
| diabetes (N=5) ^3,7,25-27^ |
| dyslipidaemia (N=5) ^12,17,19,28,29^ |
| gait impairment (N=5) ^6,30-33^ |
| dementia (N=4) ^22,24,30,34^ |
| renal impairment (N=4) ^23,35-37^ |
| stroke (N=4) ^6,13,21,38^ |
| Alzheimer's disease (N=3) ^30,39,40^ |
| depression (N=3) ^6,22,30^ |
| genetic influences (N=3) ^41-43^ |
| smoking (N=3) ^6,7,12^ |
| arterial stiffness (N=2) ^44,45^ |
| β-amyloid (Aβ) cerebral burden (N=2) ^44,46^ |
| bradykinesia (N=2) ^47^ |
| body mass index (N=2) ^3,7^ |
| cardiovascular disease (N=2) ^4,48^ |
| dietary salt consumption (N=2) ^11,49^ |
| functional outcome after stroke poor (N=2) ^50,51^ |
| intracranial atherosclerosis (N=2) ^6,52^ |
| retinal microvasculopathy (N=2) ^42,53^ |
| systemic lupus erythematosus (N=2) ^54,55^ |
| α-Klotho (anti-aging protein) reduced blood levels ^56^ |
| ANCA-associated vasculitis ^57^ |
| blood-brain barrier permeability ^58^ |
| cerebral microbleeds ^59^ |
| copper serum levels ^26^ |
| dialysis ^21^ |
| early life factors (lower age-11 IQ) ^60^ |
| fabry disease ^61^ |
| functional decline (Barthel Index) ^62^ |
| hearing loss sudden sensorineural ^63^ |
| hemoglobin level ^64^ |
| homocysteine level ^65^ |
| hemorrhage intracerebral ^66^ |
| inflammation serum parameters ^7^ |
| metabolic syndrome ^17^ |
| n-3 polyunsaturated fatty acids ^67^ |
| obese waist circumference ^68^ |
| obstructive sleep apnea ^69^ |
| prediabetes ^25^ |
| rigidity ^47^ |
| stress ^17^ |
| telomere length reduction ^18^ |

1 Fiorini, N. *et al.* Best Match: New relevance search for PubMed. *PLoS Biol* **16**, e2005343, doi:10.1371/journal.pbio.2005343 (2018).

2 Boden, J. M., Fergusson, D. M. & Horwood, L. J. Cigarette smoking and depression: tests of causal linkages using a longitudinal birth cohort. *Br J Psychiatry* **196**, 440-446, doi:10.1192/bjp.bp.109.065912 (2010).

3 Chahine, L. M. *et al.* Modifiable vascular risk factors, white matter disease and cognition in early Parkinson's disease. *Eur J Neurol* **26**, 246-e218, doi:10.1111/ene.13797 (2019).

4 Prins, N. D. & Scheltens, P. White matter hyperintensities, cognitive impairment and dementia: an update. *Nat Rev Neurol* **11**, 157-165, doi:10.1038/nrneurol.2015.10 (2015).

5 Smith, E. E. *et al.* Prevention of Stroke in Patients With Silent Cerebrovascular Disease: A Scientific Statement for Healthcare Professionals From the American Heart Association/American Stroke Association. *Stroke* **48**, e44-e71, doi:10.1161/str.0000000000000116 (2017).

6 de Havenon, A., Meyer, C., McNally, J. S., Alexander, M. & Chung, L. Subclinical Cerebrovascular Disease: Epidemiology and Treatment. *Curr Atheroscler Rep* **21**, 39, doi:10.1007/s11883-019-0799-1 (2019).

7 Lampe, L. *et al.* Visceral obesity relates to deep white matter hyperintensities via inflammation. *Ann Neurol* **85**, 194-203, doi:10.1002/ana.25396 (2019).

8 van Middelaar, T. *et al.* Effect of Antihypertensive Medication on Cerebral Small Vessel Disease: A Systematic Review and Meta-Analysis. *Stroke* **49**, 1531-1533, doi:10.1161/strokeaha.118.021160 (2018).

9 Nam, K. W. *et al.* Cerebral Small Vessel Disease and Stage 1 Hypertension Defined by the 2017 American College of Cardiology/American Heart Association Guidelines. *Hypertension* **73**, 1210-1216, doi:10.1161/hypertensionaha.119.12830 (2019).

10 Yu, X. *et al.* Risk factors of pure leukoaraiosis and the association with preclinical carotid atherosclerosis. *Atherosclerosis* **275**, 328-332, doi:10.1016/j.atherosclerosis.2018.06.869 (2018).

11 Heye, A. K. *et al.* Blood pressure and sodium: Association with MRI markers in cerebral small vessel disease. *J Cereb Blood Flow Metab* **36**, 264-274, doi:10.1038/jcbfm.2015.64 (2016).

12 Dickie, D. A. *et al.* Vascular risk factors and progression of white matter hyperintensities in the Lothian Birth Cohort 1936. *Neurobiol Aging* **42**, 116-123, doi:10.1016/j.neurobiolaging.2016.03.011 (2016).

13 Yu, L. *et al.* Age and recurrent stroke are related to the severity of white matter hyperintensities in lacunar infarction patients with diabetes. *Clin Interv Aging* **13**, 2487-2494, doi:10.2147/cia.S184463 (2018).

14 Cannistraro, R. J. *et al.* CNS small vessel disease: A clinical review. *Neurology* **92**, 1146-1156, doi:10.1212/wnl.0000000000007654 (2019).

15 Dichgans, M. & Leys, D. Vascular Cognitive Impairment. *Circ Res* **120**, 573-591, doi:10.1161/circresaha.116.308426 (2017).

16 Wallin, A. *et al.* Update on Vascular Cognitive Impairment Associated with Subcortical Small-Vessel Disease. *J Alzheimers Dis* **62**, 1417-1441, doi:10.3233/jad-170803 (2018).

17 Mijajlović, M. D. *et al.* Post-stroke dementia - a comprehensive review. *BMC Med* **15**, 11, doi:10.1186/s12916-017-0779-7 (2017).

18 Minami, R., Takahama, S. & Yamamoto, M. Correlates of telomere length shortening in peripheral leukocytes of HIV-infected individuals and association with leukoaraiosis. *PLoS ONE* **14**, e0218996, doi:10.1371/journal.pone.0218996 (2019).

19 Ke, D., Zhou, F., Liang, H., Xu, Y. & Lou, H. Hypertriglyceridemia Is Associated with Reduced Leukoaraiosis Severity in Patients with a Small Vessel Stroke. *Behav Neurol* **2018**, 1361780, doi:10.1155/2018/1361780 (2018).

20 Jiménez-Balado, J. *et al.* Cognitive Impact of Cerebral Small Vessel Disease Changes in Patients With Hypertension. *Hypertension* **73**, 342-349, doi:10.1161/hypertensionaha.118.12090 (2019).

21 Findlay, M. D. *et al.* Investigating the Relationship between Cerebral Blood Flow and Cognitive Function in Hemodialysis Patients. *J Am Soc Nephrol* **30**, 147-158, doi:10.1681/asn.2018050462 (2019).

22 Puzo, C. *et al.* Independent effects of white matter hyperintensities on cognitive, neuropsychiatric, and functional decline: a longitudinal investigation using the National Alzheimer's Coordinating Center Uniform Data Set. *Alzheimers Res Ther* **11**, 64, doi:10.1186/s13195-019-0521-0 (2019).

23 Hirao, K. *et al.* Association of serum cystatin C with white matter abnormalities in patients with amnestic mild cognitive impairment. *Geriatr Gerontol Int* **19**, 1036-1040, doi:10.1111/ggi.13767 (2019).

24 Hilal, S. *et al.* Intracranial stenosis in cognitive impairment and dementia. *J Cereb Blood Flow Metab* **37**, 2262-2269, doi:10.1177/0271678x16663752 (2017).

25 van Agtmaal, M. J. M. *et al.* Prediabetes Is Associated With Structural Brain Abnormalities: The Maastricht Study. *Diabetes Care* **41**, 2535-2543, doi:10.2337/dc18-1132 (2018).

26 Silbert, L. C. *et al.* Risk Factors Associated with Cortical Thickness and White Matter Hyperintensities in Dementia Free Okinawan Elderly. *J Alzheimers Dis* **63**, 365-372, doi:10.3233/jad-171153 (2018).

27 Schneider, A. L. C. *et al.* Diabetes, Prediabetes, and Brain Volumes and Subclinical Cerebrovascular Disease on MRI: The Atherosclerosis Risk in Communities Neurocognitive Study (ARIC-NCS). *Diabetes Care* **40**, 1514-1521, doi:10.2337/dc17-1185 (2017).

28 Todate, Y. *et al.* High Prevalence of Cerebral Small Vessel Disease on 7T Magnetic Resonance Imaging in Familial Hypercholesterolemia. *J Atheroscler Thromb* **26**, 1045-1053, doi:10.5551/jat.48553 (2019).

29 Yin, Z. G. *et al.* Sex differences in associations between blood lipids and cerebral small vessel disease. *Nutr Metab Cardiovasc Dis* **28**, 28-34, doi:10.1016/j.numecd.2017.10.001 (2018).

30 Mozumder, M. *et al.* Quantitative histomorphometry of capillary microstructure in deep white matter. *Neuroimage Clin* **23**, 101839, doi:10.1016/j.nicl.2019.101839 (2019).

31 Loos, C. M. *et al.* The relation between total cerebral small vessel disease burden and gait impairment in patients with minor stroke. *Int J Stroke* **13**, 518-524, doi:10.1177/1747493017730780 (2018).

32 Wan, Y. *et al.* Exploring the association between Cerebral small-vessel diseases and motor symptoms in Parkinson's disease. *Brain Behav* **9**, e01219, doi:10.1002/brb3.1219 (2019).

33 Pinter, D. *et al.* Impact of small vessel disease in the brain on gait and balance. *Sci Rep* **7**, 41637, doi:10.1038/srep41637 (2017).

34 Romero-Sevilla, R. *et al.* Vascular Risk Factors and Lesions of Vascular Nature in Magnetic Resonance as Predictors of Progression to Dementia in Patients with Mild Cognitive Impairment. *Curr Alzheimer Res* **15**, 671-678, doi:10.2174/1567205015666180119100840 (2018).

35 Georgakis, M. K., Chatzopoulou, D., Tsivgoulis, G. & Petridou, E. T. Albuminuria and Cerebral Small Vessel Disease: A Systematic Review and Meta-Analysis. *J Am Geriatr Soc* **66**, 509-517, doi:10.1111/jgs.15240 (2018).

36 Weiner, D. E. *et al.* Cognitive Function and Kidney Disease: Baseline Data From the Systolic Blood Pressure Intervention Trial (SPRINT). *Am J Kidney Dis* **70**, 357-367, doi:10.1053/j.ajkd.2017.04.021 (2017).

37 Makin, S. D., Cook, F. A., Dennis, M. S. & Wardlaw, J. M. Cerebral small vessel disease and renal function: systematic review and meta-analysis. *Cerebrovasc Dis* **39**, 39-52, doi:10.1159/000369777 (2015).

38 Jiang, J. *et al.* Total MRI burden of cerebral vessel disease correlates with the progression in patients with acute single small subcortical strokes. *Brain Behav* **9**, e01173, doi:10.1002/brb3.1173 (2019).

39 Salvadó, G. *et al.* Spatial patterns of white matter hyperintensities associated with Alzheimer's disease risk factors in a cognitively healthy middle-aged cohort. *Alzheimers Res Ther* **11**, 12, doi:10.1186/s13195-018-0460-1 (2019).

40 Lee, S. *et al.* White matter hyperintensities are a core feature of Alzheimer's disease: Evidence from the dominantly inherited Alzheimer network. *Ann Neurol* **79**, 929-939, doi:10.1002/ana.24647 (2016).

41 Identification of additional risk loci for stroke and small vessel disease: a meta-analysis of genome-wide association studies. *Lancet Neurol* **15**, 695-707, doi:10.1016/s1474-4422(16)00102-2 (2016).

42 van de Kreeke, J. A. *et al.* Retinal and Cerebral Microvasculopathy: Relationships and Their Genetic Contributions. *Invest Ophthalmol Vis Sci* **59**, 5025-5031, doi:10.1167/iovs.18-25341 (2018).

43 Lopez, L. M. *et al.* Genes from a translational analysis support a multifactorial nature of white matter hyperintensities. *Stroke* **46**, 341-347, doi:10.1161/strokeaha.114.007649 (2015).

44 Hughes, T. M. *et al.* Arterial stiffness and dementia pathology: Atherosclerosis Risk in Communities (ARIC)-PET Study. *Neurology* **90**, e1248-e1256, doi:10.1212/wnl.0000000000005259 (2018).

45 Chen, Y. *et al.* Low carotid endothelial shear stress associated with cerebral small vessel disease in an older population: A subgroup analysis of a population-based prospective cohort study. *Atherosclerosis* **288**, 42-50, doi:10.1016/j.atherosclerosis.2019.07.006 (2019).

46 Yi, H. A., Won, K. S., Chang, H. W. & Kim, H. W. Association between white matter lesions and cerebral Aβ burden. *PLoS ONE* **13**, e0204313, doi:10.1371/journal.pone.0204313 (2018).

47 Camarda, C. *et al.* Association Between Atrophy of the Caudate Nuclei, Global Brain Atrophy, Cerebral Small Vessel Disease and Mild Parkinsonian Signs in Neurologically and Cognitively Healthy Subjects Aged 45-84 Years: A Crosssectional Study. *Curr Alzheimer Res* **15**, 1013-1026, doi:10.2174/1567205015666180702111110 (2018).

48 Rabin, J. S. *et al.* Interactive Associations of Vascular Risk and β-Amyloid Burden With Cognitive Decline in Clinically Normal Elderly Individuals: Findings From the Harvard Aging Brain Study. *JAMA Neurol* **75**, 1124-1131, doi:10.1001/jamaneurol.2018.1123 (2018).

49 Makin, S. D. J. *et al.* Small Vessel Disease and Dietary Salt Intake: Cross-Sectional Study and Systematic Review. *J Stroke Cerebrovasc Dis* **26**, 3020-3028, doi:10.1016/j.jstrokecerebrovasdis.2017.08.004 (2017).

50 Valdés Hernández, M. D. C. *et al.* Association between Striatal Brain Iron Deposition, Microbleeds and Cognition 1 Year After a Minor Ischaemic Stroke. *Int J Mol Sci* **20**, doi:10.3390/ijms20061293 (2019).

51 Yang, C. M. *et al.* Leukoaraiosis and risk of intracranial hemorrhage and outcome after stroke thrombolysis. *PLoS ONE* **13**, e0196505, doi:10.1371/journal.pone.0196505 (2018).

52 Zhai, F. F. *et al.* Intracranial Arterial Dolichoectasia and Stenosis: Risk Factors and Relation to Cerebral Small Vessel Disease. *Stroke* **49**, 1135-1140, doi:10.1161/strokeaha.117.020130 (2018).

53 Jung, N. Y. *et al.* Retinal microvasculature changes in amyloid-negative subcortical vascular cognitive impairment compared to amyloid-positive Alzheimer's disease. *J Neurol Sci* **396**, 94-101, doi:10.1016/j.jns.2018.10.025 (2019).

54 Yeoh, H. *et al.* Relationship between cerebral microbleeds and white matter MR hyperintensities in systemic lupus erythematosus: a retrospective observational study. *Neuroradiology* **61**, 265-274, doi:10.1007/s00234-018-2130-1 (2019).

55 Wiseman, S. J. *et al.* Cerebral Small Vessel Disease Burden Is Increased in Systemic Lupus Erythematosus. *Stroke* **47**, 2722-2728, doi:10.1161/strokeaha.116.014330 (2016).

56 Kuriyama, N. *et al.* Association between α-Klotho and Deep White Matter Lesions in the Brain: A Pilot Case Control Study Using Brain MRI. *J Alzheimers Dis* **61**, 145-155, doi:10.3233/jad-170466 (2018).

57 Tani, H. *et al.* Occurrence of cerebral small vessel disease at diagnosis of MPO-ANCA-associated vasculitis. *J Neurol* **266**, 1708-1715, doi:10.1007/s00415-019-09318-9 (2019).

58 Wolters, F. J. *et al.* Cerebral Perfusion and the Risk of Dementia: A Population-Based Study. *Circulation* **136**, 719-728, doi:10.1161/circulationaha.117.027448 (2017).

59 Shoamanesh, A. *et al.* Microbleeds in the Secondary Prevention of Small Subcortical Strokes Trial: Stroke, mortality, and treatment interactions. *Ann Neurol* **82**, 196-207, doi:10.1002/ana.24988 (2017).

60 Field, T. S. *et al.* Early life characteristics and late life burden of cerebral small vessel disease in the Lothian Birth Cohort 1936. *Aging (Albany NY)* **8**, 2039-2061, doi:10.18632/aging.101043 (2016).

61 Körver, S., Vergouwe, M., Hollak, C. E. M., van Schaik, I. N. & Langeveld, M. Development and clinical consequences of white matter lesions in Fabry disease: a systematic review. *Mol Genet Metab* **125**, 205-216, doi:10.1016/j.ymgme.2018.08.014 (2018).

62 Dhamoon, M. S. *et al.* Periventricular White Matter Hyperintensities and Functional Decline. *J Am Geriatr Soc* **66**, 113-119, doi:10.1111/jgs.15149 (2018).

63 Fusconi, M. *et al.* Is there a relation between sudden sensorineural hearing loss and white matter lesions? *Eur Arch Otorhinolaryngol* **276**, 3043-3049, doi:10.1007/s00405-019-05593-4 (2019).

64 Wolters, F. J. *et al.* Hemoglobin and anemia in relation to dementia risk and accompanying changes on brain MRI. *Neurology* **93**, e917-e926, doi:10.1212/wnl.0000000000008003 (2019).

65 Fan, H. *et al.* Study on the incidence and risk factor of silent cerebrovascular disease in young adults with first-ever stroke. *Medicine (Baltimore)* **97**, e13311, doi:10.1097/md.0000000000013311 (2018).

66 Park, Y. S., Chung, M. S. & Choi, B. S. MRI Assessment of Cerebral Small Vessel Disease in Patients with Spontaneous Intracerebral Hemorrhage. *Yonsei Med J* **60**, 774-781, doi:10.3349/ymj.2019.60.8.774 (2019).

67 Bowman, G. L. *et al.* Randomized Trial of Marine n-3 Polyunsaturated Fatty Acids for the Prevention of Cerebral Small Vessel Disease and Inflammation in Aging (PUFA Trial): Rationale, Design and Baseline Results. *Nutrients* **11**, doi:10.3390/nu11040735 (2019).

68 Arnoldussen, I. A. C., Gustafson, D. R., Leijsen, E. M. C., de Leeuw, F. E. & Kiliaan, A. J. Adiposity is related to cerebrovascular and brain volumetry outcomes in the RUN DMC study. *Neurology* **93**, e864-e878, doi:10.1212/wnl.0000000000008002 (2019).

69 Ho, B. L. *et al.* Obstructive sleep apnea and cerebral white matter change: a systematic review and meta-analysis. *J Neurol* **265**, 1643-1653, doi:10.1007/s00415-018-8895-7 (2018).

**Study Design/Subject selection:**

KORA MRI comprises 400 participants and is a sub-study of the population-based KORA FF4 study (2013-2014, 2279 subjects). The FF4 study is the second follow-up of the baseline study KORA S4 (1999-2001, 4261 subjects), which is a large sample from the general population in the region of Augsburg, Germany. Exclusion criteria for the MRI sub-study were: age >74 years, validated/self-reported stroke, myocardial infarction, peripheral artery disease (PAD), type 1 diabetes mellitus, poor overall health condition, missing oral glucose tolerance test result (OGTT) or contraindications to MRI [1].

The MRI-data was originally acquired as a nested case-control study to investigate differences in subclinical cardiovascular disease between persons with diabetes, prediabetes and non-diabetic controls. Consequently, the MRI sub-study was enriched with prediabetic and diabetic individuals. However, additional analyses for dissimilarities between the aforementioned MRI sub-study and the whole KORA-cohort did not reveal any differences [2].

SHIP-TREND-0 is a population-based cohort sampled from the population of West Pomerania in North Eastern Germany [3]. Participants in the age range of 20 to 79 years were recruited by a random cluster sample, and 4 420 individuals were included in SHIP-TREND-0. Standardized interviews and examinations were conducted between 2008 and 2012. Individuals eligible and willing to undergo whole-body MRI participated in the MRI substudy**.**

1 Bamberg F, Hetterich H, Rospleszcz S et al (2017) Subclinical Disease Burden as Assessed by Whole-Body MRI in Subjects With Prediabetes, Subjects With Diabetes, and Normal Control Subjects From the General Population: The KORA-MRI Study. Diabetes 66:158-169

2 Rospleszcz S, Schafnitzel A, Koenig W et al (2018) Association of glycemic status and segmental left ventricular wall thickness in subjects without prior cardiovascular disease: a cross-sectional study. BMC Cardiovascular Disorders 18:162

3 Völzke H, Alte D, Schmidt CO et al (2011) Cohort profile: the study of health in Pomerania. International Journal of Epidemiology 40:294-307

**Supplementary Table II:**

|  | Description |
| --- | --- |
| **Sociodemographics** | **Further reference: [1-4]** |
| Age, years | Self-reported in standardized interview |
| Family status | Self-reported in standardized interview |
| Schooling | Self-reported in standardized interview |
| Schooling, years | calculated based on self-report in standardized interview |
| Highest professional degree | Self-reported in standardized interview |
| Per-capita income, Euro | calculated based on self-report in standardized interview |
| Equivalence income, Euro | calculated based on per-capita income, weighted according to number and age of all household members. Weights are derived according to cost of living, following Bundessozialhilfegesetz (BSHG) |
| Social stratum, Helmert scale | numeric score based on schooling, degree, job position and equivalence income |
| **Anthropometric measurements** | **Further reference: [5]** |
| Weight, kg | measured in standardized examination by calibrated steelyards or digital scales (SECA 635 or SECA 877 or SECA measuring station 285, Seca GmbH & Co, KG, Hamburg, Germany) |
| Height, cm | measured in standardized examination by calibrated levelling bar (SECA 242, Seca GmbH & Co, KG, Hamburg, Germany) |
| BMI, kg/m2 | calculated as weight in kg divided by squared height in m |
| Waist circumference, cm | measured in standardized examination with an inelastic tape at the level midway between the lower rib margin and the iliac crest |
| Hip circumference, cm | measured in standardized examination with an inelastic tape at the level of maximal gluteal protrusion |
| Waist-To-Hip Ratio | calculated as waist circumference in cm divided by hip circumference in cm |
| right-handed | Self-reported in standardized interview |
| **Other metabolic measurements** | **Further reference: [6-8]** |
| Hepatic Fat, % | MRI measurement: proton density fat fraction by multiecho single-voxel 1H spectroscopy |
| Visceral Fat, l | MRI measurement: calculated semiautomatically from volume-interpolated three-dimensional in/opposed-phase volumetric interpolated Dixon sequence from femoral head to the diaphragm |
| **Diabetes related measurements** | **Further reference: [4, 9, 10]** |
| Glycemic Status | determined as either established type-2 diabetes (validated by physician) or after OGTT according to WHO criteria. OGTT was based on 300ml of liquid containing 75g of carbohydrates. |
| normal | fasting glucose < 110 mg/dL and 2h glucose < 140 mg/dL |
| prediabetes | 110 mg/dL <= fasting glucose <= 125 mg/dL and/or 140 mg/dL <= 2-h glucose <= 200 mg/dL |
| diabetes | fasting glucose > 140 mg/dL and/or 2-h glucose > 200 mg/dL |
| Duration of diabetes, years | calculated based on self report |
| Fasting glucose, mg/dL (Serum) | UV test using enzymatic reference method with hexokinase (Vista, Siemens or Cobas, Roche) |
| Fasting insulin, mg/dL (Serum) | Elecsys Insulin immunoassay with two monoclonal antibodies (Vista, Siemens or Cobas, Roche) |
| HbA1c, % (hemolyzed whole blood) | cation-exchange high performance liquid chromatographic, photometric assay (VARIANT II TURBO Hemoglobon Testing System, Bio-Rad Laboratories Inc, Hercules, US) |
| **Behavioural factors** | **Further reference: [11-13]** |
| Alcohol consumption, categorical or g/day | calculated based on self-reported amount and type of alcoholic beverages consumed |
| Smoking | Self-reported in standardized interview |
| Packyears | calculated based on self-reported number of cigarettes smoked |
| Physically active | Self-reported in standardized interview |
| Physical activity | calculated based on self-report in standardized interview |
| **Somatic and Depressive Symptoms** | **Further references: [14-16]** |
| Angina Pectoris | determined based on self-reported symptoms in standardized interview |
| Sf-12 Somatic Scale | determined based on standardized questionnaire |
| Depressive Symptoms: PHQ-9 | assessed using the 9-item Patient Health Questionnaire (PHQ-9) in standardized interview |
| Depressed mood/Exhaustion: DEEX scale | Assessed using the Depression and Exhaustion (DEEX) scale consisting of eight items with a 4-point scale |
| **Medication intake** | **Further reference: [17]** |
| Antidiabetic | based on standardized interview. Participants were asked to bring packages of every medication that they had taken in the 7 days before the interview. Additionally, medication intake was assessed by interview.  ATC Codes A10 |
| Antihypertensive | compounds from ATC Codes C02, C03, C07, C08, C09 when German guidelines classify the compound as anti-hypertensive |
| Anticoagulant | ATC Codes B01AA, B01AB, B01AE, B01AF, B01AX |
| Antiplatelet | ATC Codes B01AC |
| Thyroidal | ATC Codes H03 (but not H03PB, H03BP, H03CA) |
| NSAID | ATC Codes N02B or M01A (but not M01AX), R05XA, N02AA59, N02AA69, N02AX62 |
| ASS 100/300 | ATC Codes B01AC06 |
| **Blood pressure** | **Further reference: [18]** |
| Systolic BP, mmHg | 3 measurements with an oscillometric digital device (OMRON HEM-705CP). Average of 2nd and 3rd measurements. |
| Diastolic BP, mmHg | 3 measurements with an oscillometric digital device (OMRON HEM-705CP). Average of 2nd and 3rd measurements. |
| Pulse Pressure | 3 measurements with an oscillometric digital device (OMRON HEM-705CP). Average of 2nd and 3rd measurements. |
| Hypertension | defined as systolic/diastolic blood pressure above 140/90 mmHg or intake of antihypertensive medication, given that the participant was aware of being hypertensive. |
| Control and awareness of hypertension | based on blood pressure measurements as detailed above, self-reported diagnosis of hypertension by a physician and intake of antihypertensive medication |
| Hypertension, controlled | Participant is normotensive due to hypertension treatment, and aware of having hypertension |
| Hypertension, uncontrolled | Participant is hypertensive, treated for hypertension, and aware of having hypertension |
| Hypertension, untreated | Participant is hypertensive, not treated for hypertension, and aware of having hypertension |
| Hypertension, unknown | Participant is hypertensive and unaware of having hypertension |
| **Sleep** | **Further reference: [19]** |
| Sleep, h/day | Self-reported in standardized interview |
| Problems falling asleep | Self-reported in standardized interview |
| Problems keeping asleep | Self-reported in standardized interview |
| Feeling tired and exhausted because of sleep problems | Self-reported in standardized interview |
| **Laboratory values** | **Further reference: [10, 20]** |
| Glomerular Filtration Rate | sex-specific calculation based on serum creatinine according to CKD-EPI |
| Total cholesterol, mg/dL (Serum) | Enzymatic, colorimetric CHOL Flex assay (Vista, Siemens or Cobas, Roche) |
| HDL cholesterol, mg/dL (Serum) | Enzymatic, colorimetric LDLC Flex assay (Vista, Siemens or Cobas, Roche) |
| LDL cholesterol, mg/dL (Serum) | Enzymatic, colorimetric HDLC Flex assay (Vista, Siemens or Cobas, Roche) |
| Triglycerides, mg/dL (Serum) | Enzymatic, colorimetric TRIG Flex assay (Vista, Siemens or Cobas, Roche) |
| Uric Acid, mg/dL (Serum) | Enzymatic colorimetric UA Flex assay (Vista, Siemens or Cobas, Roche) |
| Creatinine, mg/dL (Serum) | Kinetic colorimetric CREJ assay based on Jaffé method |

**Supplementary Table II:** Description of extracranial parameters including measures of diabetes, blood pressure, adipose tissue, medication intake, sociodemographics, anthropometrics, behaviour, somatic and depressive symptoms and sleep. Extracranial parameters were collected in a standardized method as part of the KORA study design. Please note that although the reference might not pertain to KORA FF4 but to one of the other KORA surveys, the described procedure was also applicable in FF4.

1. Helmert U: **Soziale Ungleichheit und Krankheitsrisiken**: MaroVerl.; 2003.

2. Holle R, Happich M, Löwel H, Wichmann H: **KORA--a research platform for population based health research**. *Gesundheitswesen (Bundesverband der Arzte des Offentlichen Gesundheitsdienstes (Germany))* 2005, **67**:S19-25.

3. Rathmann W, Haastert B, Icks A, Giani G, Holle R, Meisinger C, Mielck A: **Sex differences in the associations of socioeconomic status with undiagnosed diabetes mellitus and impaired glucose tolerance in the elderly population: the KORA Survey 2000**. *The European Journal of Public Health* 2005, **15**(6):627-633.

4. Rathmann W, Haastert B, Icks A, Lowel H, Meisinger C, Holle R, Giani G: **High prevalence of undiagnosed diabetes mellitus in Southern Germany: target populations for efficient screening. The KORA survey 2000**. *Diabetologia* 2003, **46**(2):182-189.

5. Lorbeer R, Rospleszcz S, Schlett CL, Heber SD, Machann J, Thorand B, Meisinger C, Heier M, Peters A, Bamberg F: **Correlation of MRI-derived adipose tissue measurements and anthropometric markers with prevalent hypertension in the community**. *Journal of hypertension* 2018, **36**(7):1555-1562.

6. Bamberg F, Hetterich H, Rospleszcz S, Lorbeer R, Auweter SD, Schlett CL, Schafnitzel A, Bayerl C, Schindler A, Saam T *et al*: **Subclinical Disease Burden as Assessed by Whole-Body MRI in Subjects With Prediabetes, Subjects With Diabetes, and Normal Control Subjects From the General Population: The KORA-MRI Study**. *Diabetes* 2017, **66**(1):158-169.

7. Hetterich H, Bayerl C, Peters A, Heier M, Linkohr B, Meisinger C, Auweter S, Kannengießer SA, Kramer H, Ertl-Wagner B: **Feasibility of a three-step magnetic resonance imaging approach for the assessment of hepatic steatosis in an asymptomatic study population**. *European radiology* 2016, **26**(6):1895-1904.

8. Storz C, Heber SD, Rospleszcz S, Machann J, Sellner S, Nikolaou K, Lorbeer R, Gatidis S, Elser S, Peters A: **The role of visceral and subcutaneous adipose tissue measurements and their ratio by magnetic resonance imaging in subjects with prediabetes, diabetes and healthy controls from a general population without cardiovascular disease**. *The British journal of radiology* 2018, **91**(xxxx):20170808.

9. Kowall B, Rathmann W, Stang A, Bongaerts B, Kuss O, Herder C, Roden M, Quante A, Holle R, Huth C *et al*: **Perceived risk of diabetes seriously underestimates actual diabetes risk: The KORA FF4 study**. *PLOS ONE* 2017, **12**(1):e0171152.

10. Laxy M, Knoll G, Schunk M, Meisinger C, Huth C, Holle R: **Quality of Diabetes Care in Germany Improved from 2000 to 2007 to 2014, but Improvements Diminished since 2007. Evidence from the Population-Based KORA Studies**. *PLoS One* 2016, **11**(10):e0164704.

11. Meisinger C, Löwel H, Thorand B, Döring A: **Leisure time physical activity and the risk of type 2 diabetes in men and women from the general population**. *Diabetologia* 2005, **48**(1):27-34.

12. Schneider B, Baumert J, Schneider A, Marten-Mittag B, Meisinger C, Erazo N, Hammer GP, Ladwig K-H: **The effect of risky alcohol use and smoking on suicide risk: findings from the German MONICA/KORA-Augsburg Cohort Study**. *Social Psychiatry and Psychiatric Epidemiology* 2011, **46**(11):1127-1132.

13. Zeilinger S, Kühnel B, Klopp N, Baurecht H, Kleinschmidt A, Gieger C, Weidinger S, Lattka E, Adamski J, Peters A *et al*: **Tobacco Smoking Leads to Extensive Genome-Wide Changes in DNA Methylation**. *PLOS ONE* 2013, **8**(5):e63812.

14. Rabel M, Meisinger C, Peters A, Holle R, Laxy M: **The longitudinal association between change in physical activity, weight, and health-related quality of life: Results from the population-based KORA S4/F4/FF4 cohort study**. *PLOS ONE* 2017, **12**(9):e0185205.

15. Kroenke K, Spitzer RL, Williams JBW: **The PHQ-9: Validity of a Brief Depression Severity Measure**. *Journal of General Internal Medicine* 2001, **16**(9):606-613.

16. Ladwig K-H, Marten-Mittag B, Baumert J, Löwel H, Döring A, Investigators K: **Case-finding for depressive and exhausted mood in the general population: reliability and validity of a symptom-driven diagnostic scale. Results from the prospective MONICA/KORA Augsburg Study**. *Annals of epidemiology* 2004, **14**(5):332-338.

17. Teuner CM, Menn P, Heier M, Holle R, John J, Wolfenstetter SB: **Impact of BMI and BMI change on future drug expenditures in adults: results from the MONICA/KORA cohort study**. *BMC Health Services Research* 2013, **13**(1):424.

18. Lorbeer R, Bayerl C, Auweter S, Rospleszcz S, Lieb W, Meisinger C, Heier M, Peters A, Bamberg F, Hetterich H: **Association between MRI-derived hepatic fat fraction and blood pressure in participants without history of cardiovascular disease**. *Journal of hypertension* 2017, **35**(4):737-744.

19. Helbig AK, Stöckl D, Heier M, Thorand B, Schulz H, Peters A, Ladwig K-H, Meisinger C: **Relationship between sleep disturbances and multimorbidity among community-dwelling men and women aged 65–93 years: results from the KORA Age Study**. *Sleep Medicine* 2017, **33**:151-159.

20. Inker LA, Schmid CH, Tighiouart H, Eckfeldt JH, Feldman HI, Greene T, Kusek JW, Manzi J, Van Lente F, Zhang YL: **Estimating glomerular filtration rate from serum creatinine and cystatin C**. *New England Journal of Medicine* 2012, **367**(1):20-29.

**Handling of missing values:**

In the KORA sample T2w 3D-FLAIR cMRI images were not assessable in 12 participants. In 16 cases hepatic or visceral fat measurements were missing. Visible lesions with other aetiology were reported in 2 cases (1 participant with lesions suspicious for multiple sclerosis; 1 participant with not WMH-like FLAIR-hyperintense lesion in the left parietal lobe).

As missing values in the predictor variables of interest were rare, they were imputed by single imputation based on predictive mean matching using 5 cases in each match set for continuous variables and logistic regression based for dichotomous variables [4]. Variables used in the imputation model were all predictor variables of interest as well as the outcome. We used R package MICE for imputation.

In SHIP, all participants with any missing values were excluded from the analysis.

4 Morris TP, White IR, Royston P (2014) Tuning multiple imputation by predictive mean matching and local residual draws. BMC medical research methodology 14:75

**Supplementary Table III:**

|  | | **KORA sample** | |  | | **SHIP sample** | |
| --- | --- | --- | --- | --- | --- | --- | --- |
| **Variable** | | **N selected** | **mean beta** | **Variable** | | **N selected** | **mean beta** |
| 1 | Age (years) | 224 | 0.349 | 1 | Age | 1000 | 0.742 |
| 2 | Hypertension, controlled | 169 | 0.305 | 2 | Hypertension, controlled | 1000 | 0.616 |
| 3 | HbA1c | 148 | 0.045 | 3 | Physical activity: 2h/week | 978 | 0.238 |
| 4 | Widowed | 145 | 0.396 | 4 | Hypertension, unknown | 966 | 0.341 |
| 5 | Prediabetes | 135 | 0.148 | 5 | NSAID medication | 906 | -0.170 |
| 6 | Medication: Antiplatelet | 134 | 0.501 | 6 | separated or divorced | 877 | -0.162 |
| 7 | Hypertension, unknown | 106 | 0.726 | 7 | living alone | 800 | 0.169 |
| 8 | Medication: NSAID | 65 | 0.716 | 8 | Prediabetes | 656 | 0.039 |
| 9 | Physical activity: 2h/week | 46 | -0.097 | 9 | Alcohol consumption: 20-40 g/day | 615 | 0.050 |
| 10 | Alcohol consumption: 1-20g/day | 46 | 0.085 | 10 | Alcohol consumption: 1-20g/day | 576 | -0.031 |

**Supplementary Table III:** Table to Figure 4. Top ten selected variables for α = 0.8 of the ZINB model based on elastic net regularization in KORA and α = 1 of the NB model in SHIP. Beta coefficient of the respective variable, averaged over all splits where the variable was selected.

**Supplementary Table IV:**

| **Rank** | **Variable** | **N** |
| --- | --- | --- |
| 1 | age | 212 |
| 2 | controlled hypertension | 198 |
| 3 | antiplatelet medication | 192 |
| 4 | alcohol consumption > 40 g/day | 182 |
| 5 | widowed | 176 |
| 6 | HbA1c | 165 |
| 7 | untreated hypertension | 154 |
| 8 | prediabetes | 143 |
| 9 | alcohol consumption | 110 |
| 10 | often problems keeping asleep | 96 |
| … | … | … |
| 23 | ICV | 18 |

**Supplementary Table IV:** Results of the elastic net regression: Selection frequencies (N) when intracranial volume (ICV) is included as predictor variable in N=333 KORA participants.

**Supplementary Figure I:**


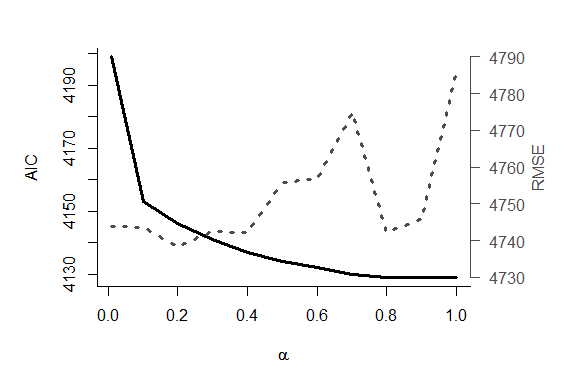


**Supplementary Figure I:** Model properties according to the grid of α-values. On the x-axis: α-value. On the left y-axis (solid line): Akaikes Information Criterion (AIC) values, averaged over 1000 splits. On the right y-axis (dotted line): Root Mean Squared Error (RMSE), averaged over 1000 splits. As both AIC and RMSE should be minimized, α=0.8 provided the best trade-off. Note that AIC of the Null Model (AIC = 1396092) and RMSE of the Null Model (RMSE = 4829) are not plotted, as they exceed the y-axes.


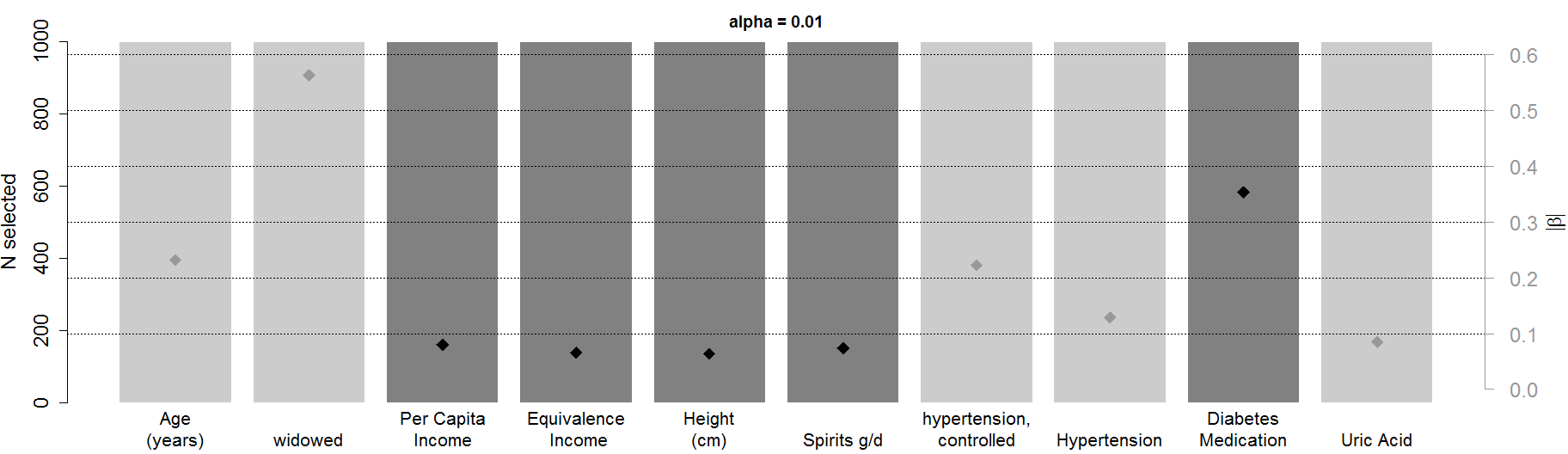
**Supplementary Figure II:**


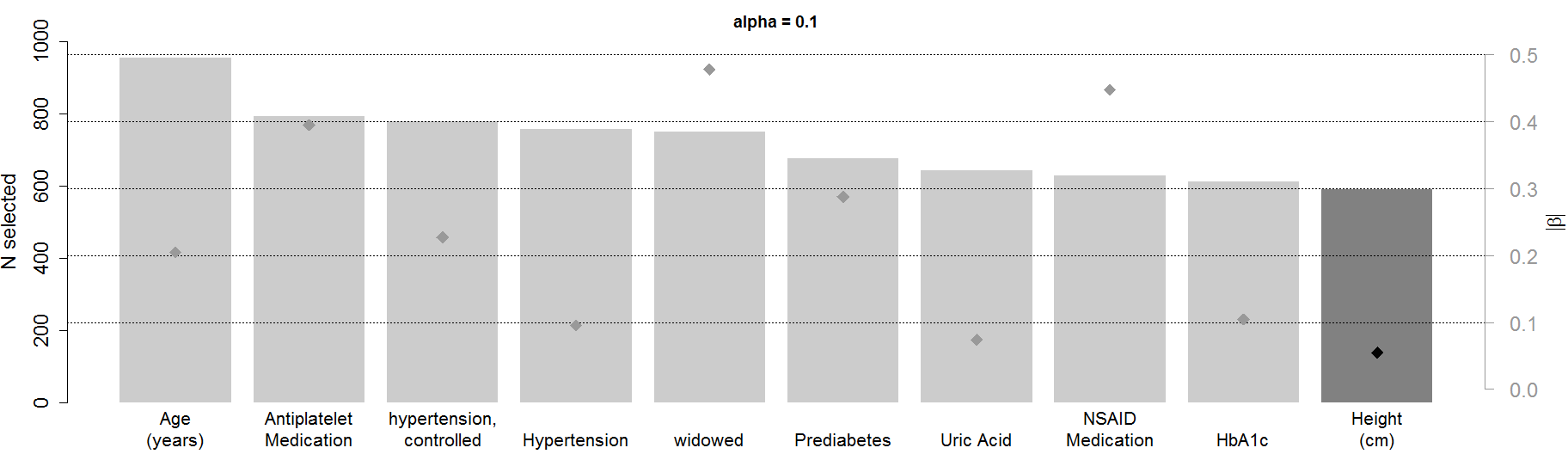


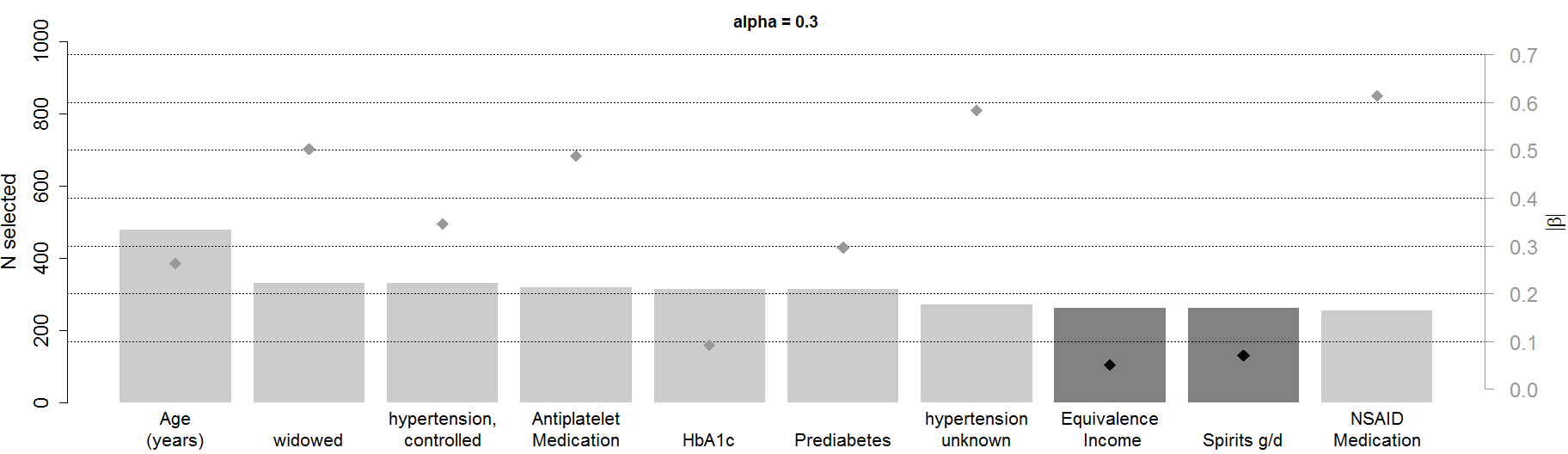

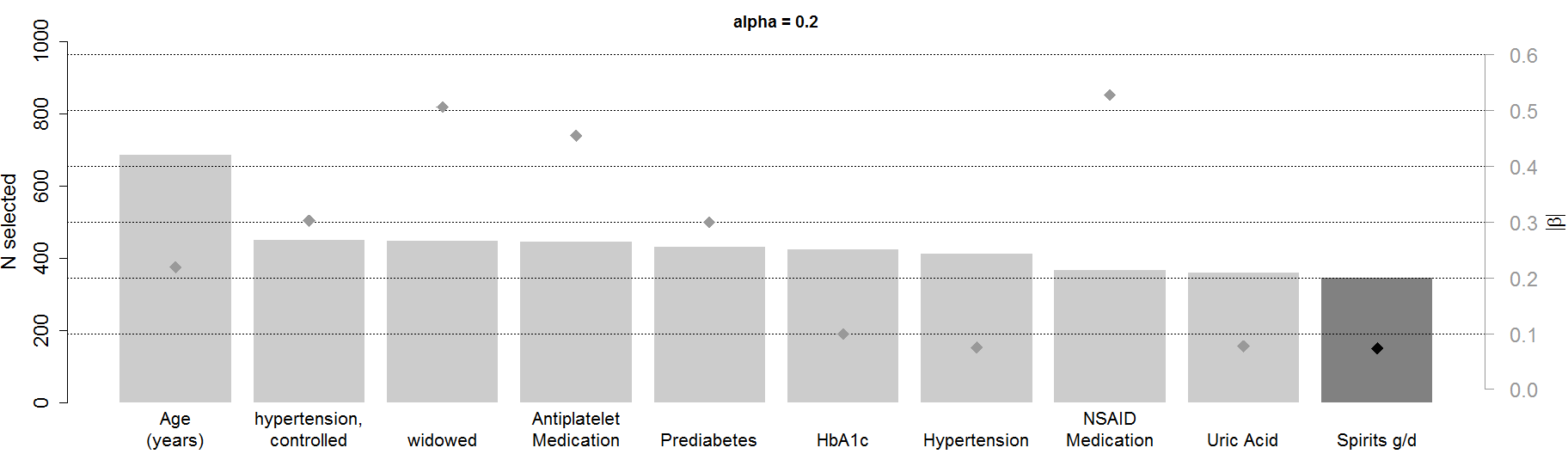


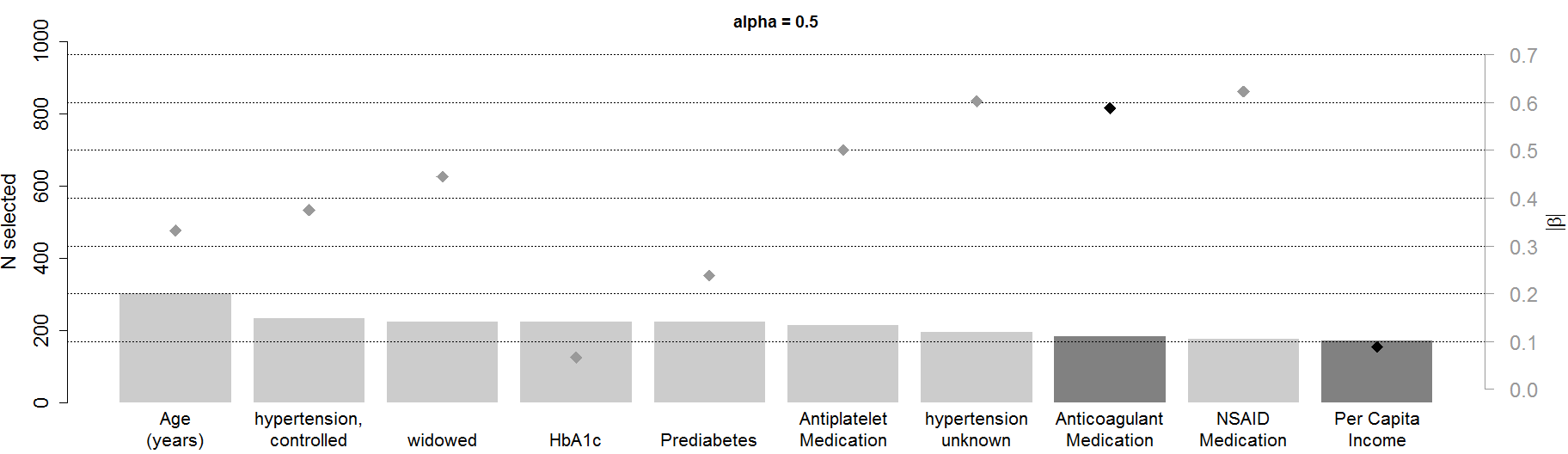

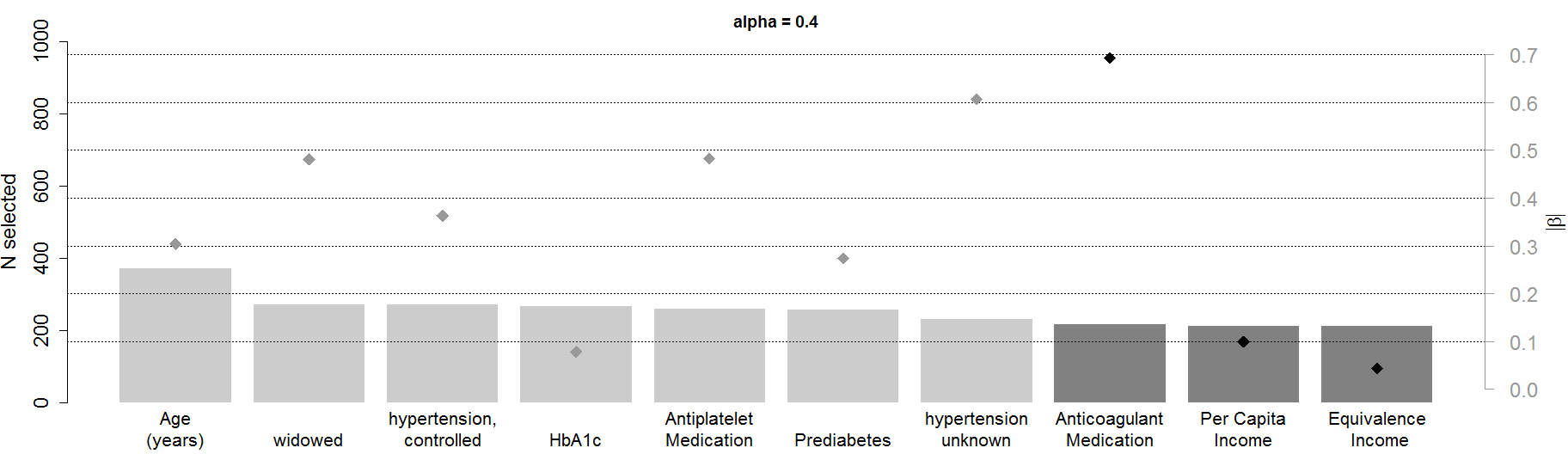


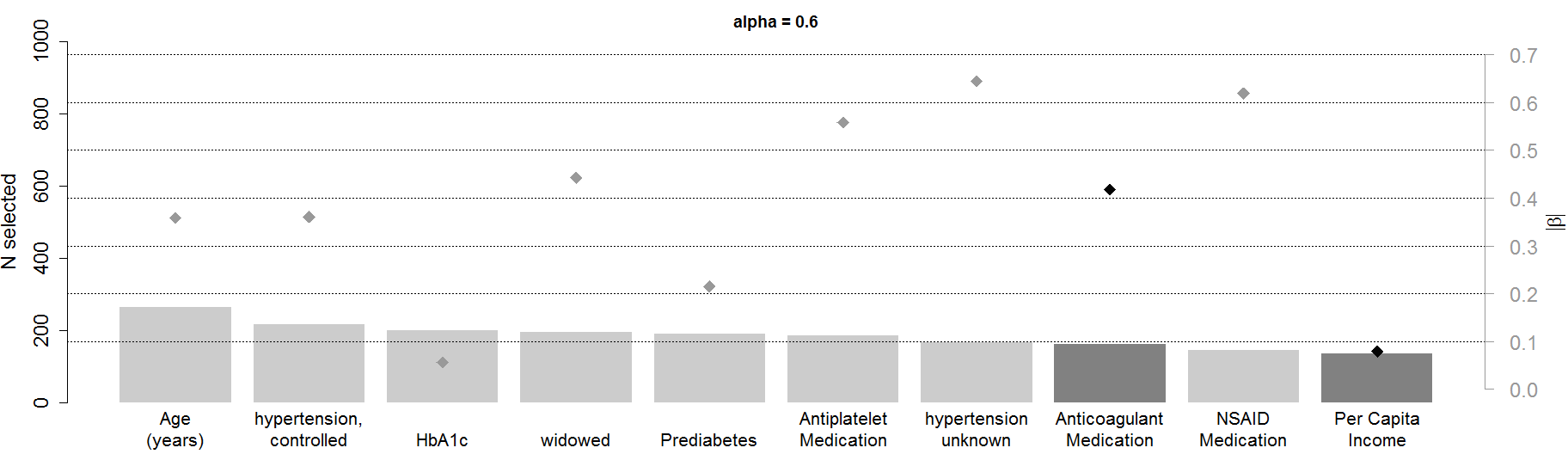

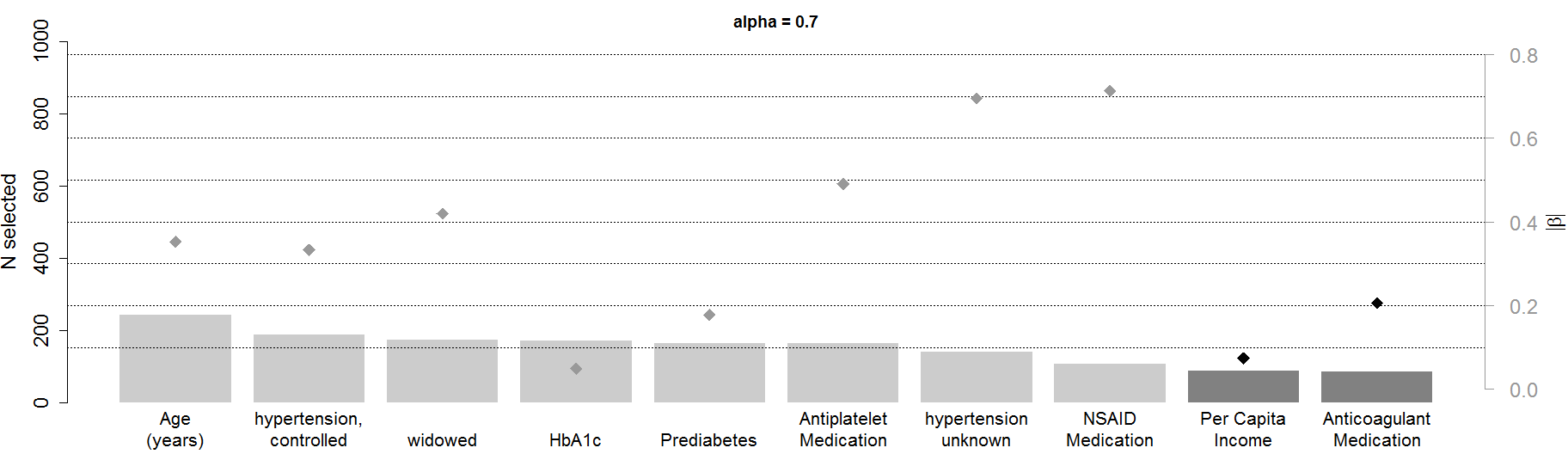


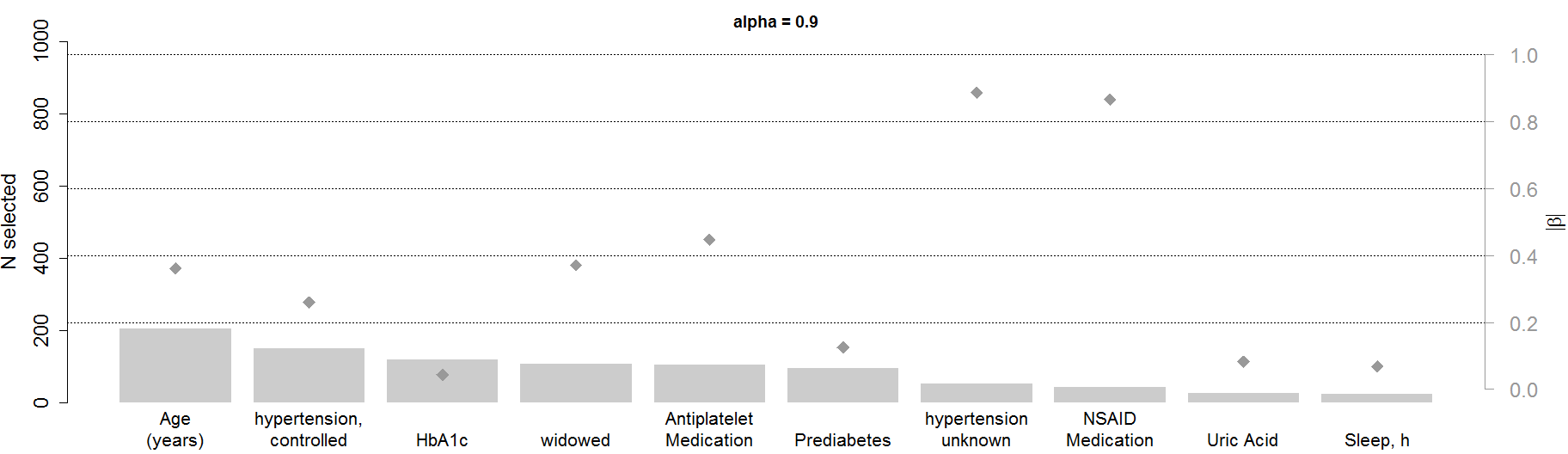

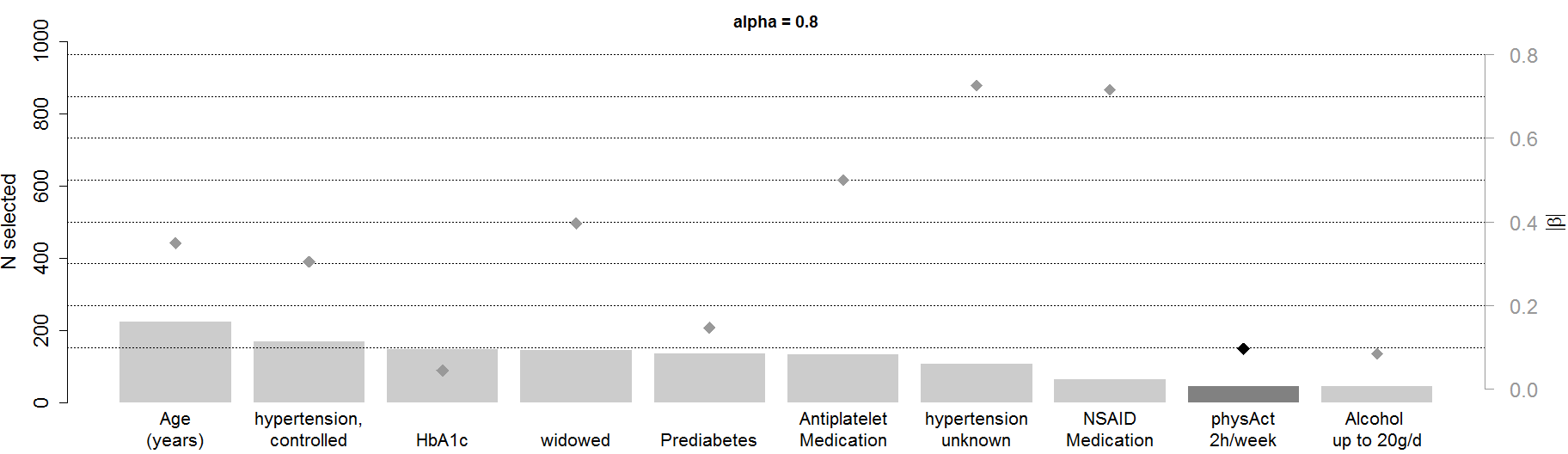


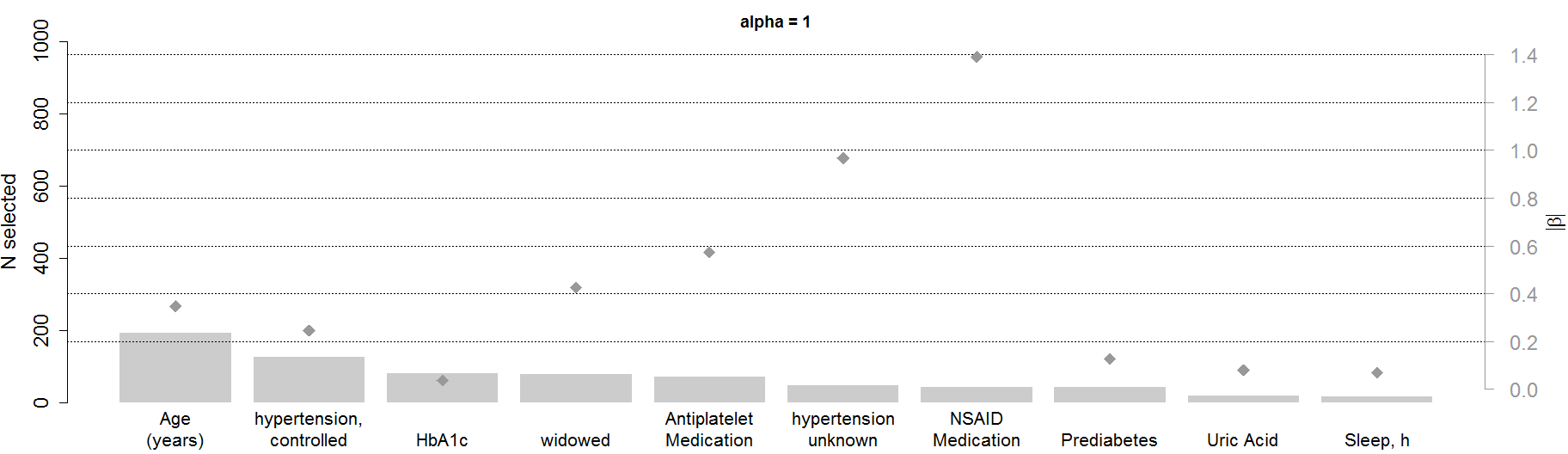


**Supplementary Figure II:** Top ten selected variables for α = 0.01 – 1.0 of the ZINB model based on elastic net regularization. On the x-axis: Predictor variable. Transparent grey bars indicate the number of data splits (of 1000) where the respective variable was selected (scale according to the left y-axis). Filled grey diamonds indicate the size of the beta coefficient of the respective variable, averaged over all splits where the variable was selected (scale according to the right y-axis). Light grey indicates a beta coefficient > 0. Dark grey indicates a beta coefficient < 0.

**Supplementary Figure III:**


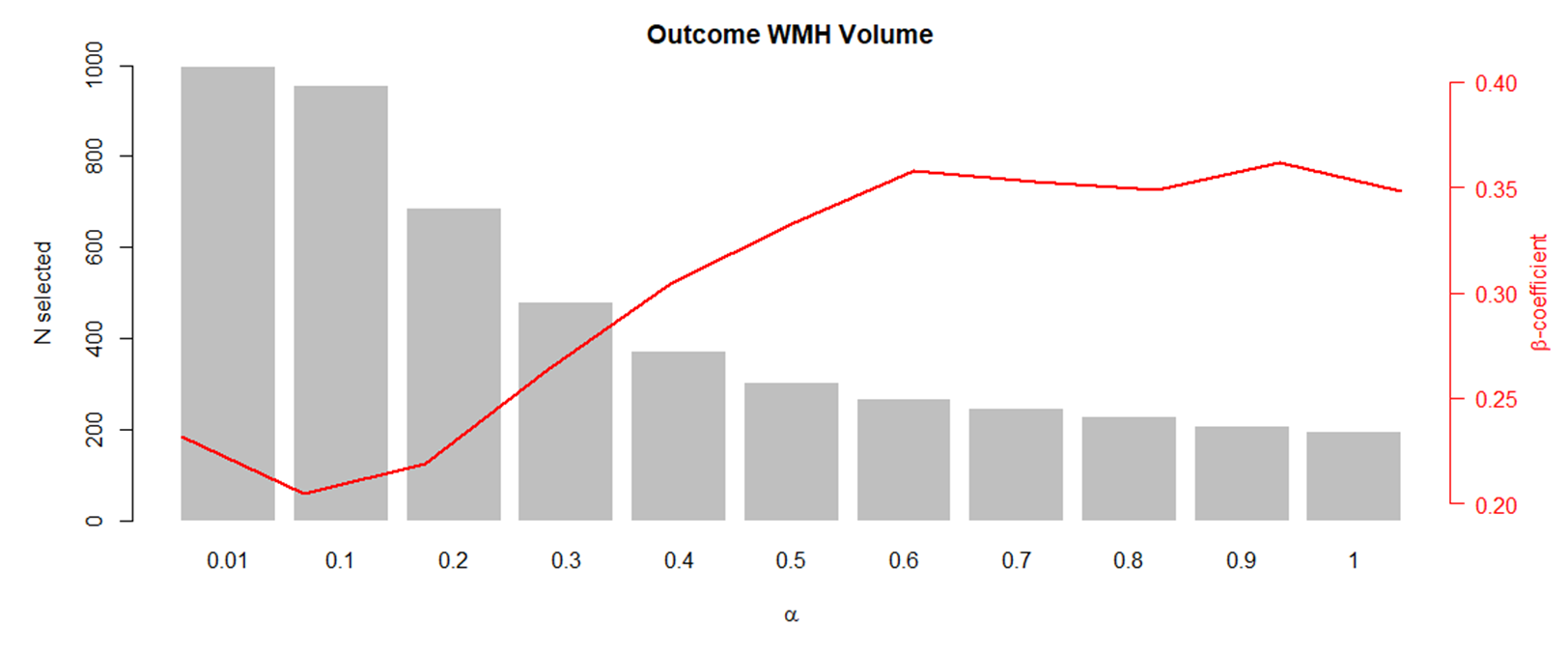


**Supplementary Figure III:** Exemplary graph how number of selected splits and β-coefficients evolve on the grid of α-values. This figure shows results for the variable age. On the x-axis: α-value. On the left y-axis (grey bars): Number of splits where variable age was selected. On the right y-axis (red lines): estimated β-coefficients of variable age.

**
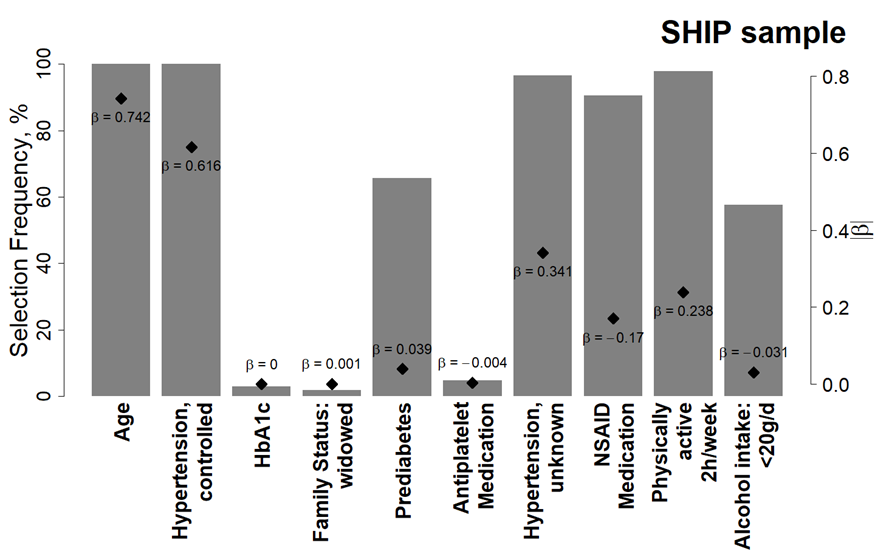
Supplementary Figure IV:**

**Supplementary Figure IV:** Selection frequencies in the SHIP sample. Results are based on a negative binomial regression model with elastic net regularization for α=1. Shown are the top ten variables that were determined in the KORA sample ranked according to selection frequency in the KORA sample. On the x-axis: Predictor variable. Grey bars indicate the selection frequency (%) of the respective variable in 1000 data splits (scale according to the left y-axis). Diamonds indicate the size of the β coefficient of the respective variable, averaged over all splits where the variable was selected (scale according to the right y-axis).

**Supplementary Figure V:**

**
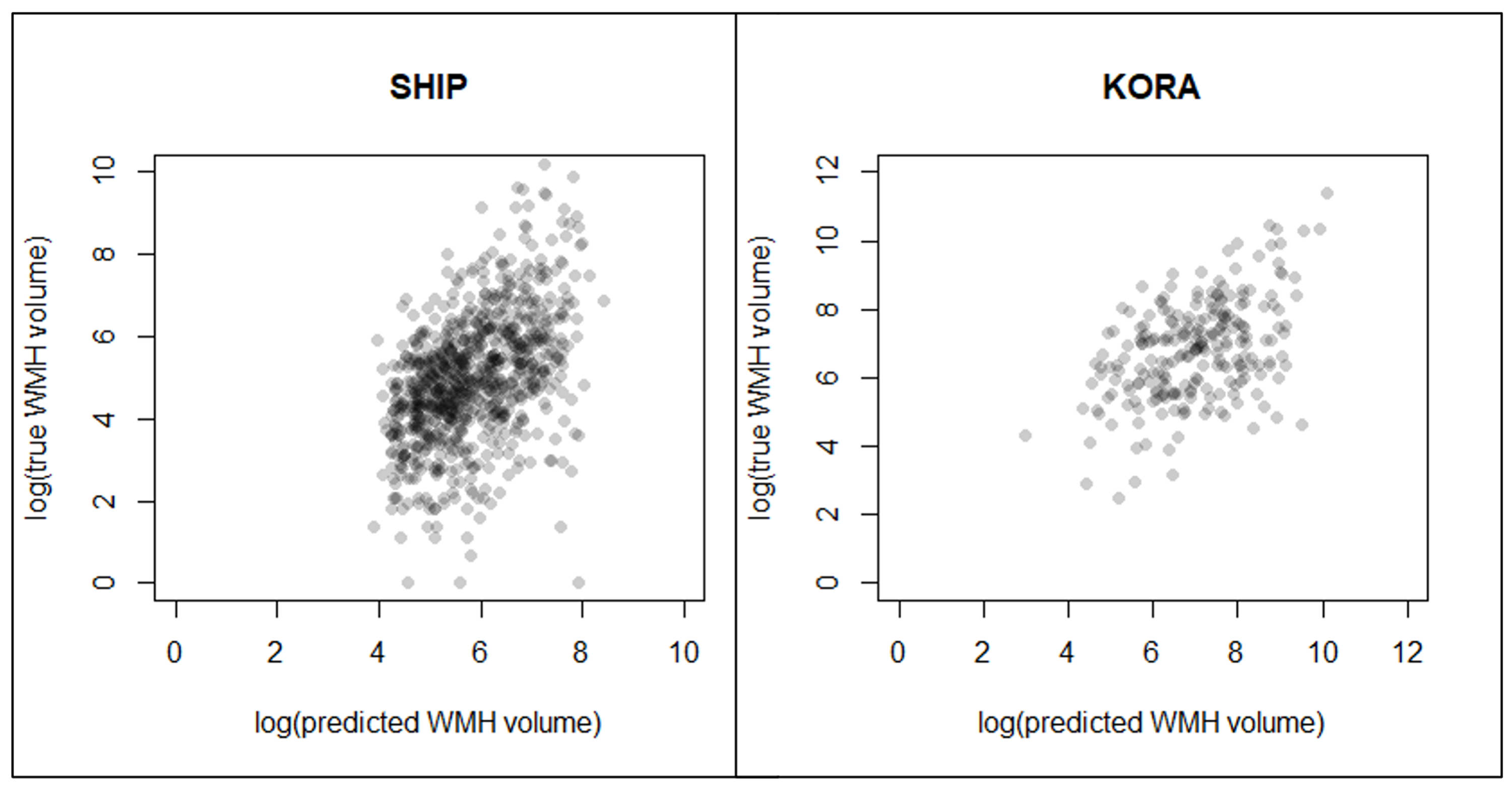
**

**Supplementary Figure V:** On the x-axis: log-transformed predicted values from the model including the top 10 variables. On the y-axis: log-transformed true WMH volumes (>0). The correlation between log-transformed predicted and log-transformed true values as measured by Spearman‘s rho was 0.48 in SHIP and 0.41 in KORA.. For log-transformed values, the RMSE amounts to 1.61 and 1.41 for the Top 10 model in SHIP and KORA, respectively, and 2.00 and 1.60 for the Null model in SHIP and KORA, respectively.

**WMH volume:**

The median WMH volumes in both samples of this study (KORA sample: median WMH volume 997 mm^3^ (mean age 56 years); SHIP sample: median WMH volume 135 mm^3^ (mean age 54 years)) are in keeping with current literature, particularly with regard to age. Van Agtmaal et al. reported in a comparable population-based cohort study of 2,228 participants a median WMH volume of 238 mm^3^ (mean age 59 years) [1]. For comparison, Schneider et al. reported a noticeably higher median WMH volume of 10,320 mm^3^ in 1,713 participants of a community-based cohort study with a markedly higher mean age of 75 years [2]. Further studies reported median WMH volumes of 1,500 mm^3^ (mean age 63 years) [3], 1,700 mm^3^ (mean age 75 years) [4], 5,600 mm^3^ (mean age 72 years) [5], and 13,300 mm^3^ (mean age 74 years) [6].

1 van Agtmaal MJM, Houben AJHM, de Wit V et al (2018) Prediabetes Is Associated With Structural Brain Abnormalities: The Maastricht Study. Diabetes Care 41:2535-2543

2 Schneider ALC, Selvin E, Sharrett AR et al (2017) Diabetes, Prediabetes, and Brain Volumes and Subclinical Cerebrovascular Disease on MRI: The Atherosclerosis Risk in Communities Neurocognitive Study (ARIC-NCS). Diabetes Care 40:1514-1521

3 Grool AM, van der Graaf Y, Mali WP, Geerlings MI (2011) Location of cerebrovascular and degenerative changes, depressive symptoms and cognitive functioning in later life: the SMART-Medea study. J Neurol Neurosurg Psychiatry 82:1093-1100

4 Versluis CE, van der Mast RC, van Buchem MA et al (2006) Progression of cerebral white matter lesions is not associated with development of depressive symptoms in elderly subjects at risk of cardiovascular disease: The PROSPER Study. International Journal of Geriatric Psychiatry 21:375-381

5 Godin O, Dufouil C, Maillard P et al (2008) White matter lesions as a predictor of depression in the elderly: the 3C-Dijon study. Biol Psychiatry 63:663-669

6 Teodorczuk A, Firbank MJ, Pantoni L et al (2010) Relationship between baseline white-matter changes and development of late-life depressive symptoms: 3-year results from the LADIS study. Psychological Medicine 40:603-610
